# Caspases in retinal ganglion cell death and axon regeneration

**DOI:** 10.1038/cddiscovery.2017.32

**Published:** 2017-07-03

**Authors:** Chloe N Thomas, Martin Berry, Ann Logan, Richard J Blanch, Zubair Ahmed

**Affiliations:** 1 Neuroscience and Ophthalmology, Institute of Inflammation and Ageing, University of Birmingham, Birmingham, UK; 2 Academic Department of Military Surgery and Trauma, Royal Centre for Defence Medicine, Birmingham, UK

## Abstract

Retinal ganglion cells (RGC) are terminally differentiated CNS neurons that possess limited endogenous regenerative capacity after injury and thus RGC death causes permanent visual loss. RGC die by caspase-dependent mechanisms, including apoptosis, during development, after ocular injury and in progressive degenerative diseases of the eye and optic nerve, such as glaucoma, anterior ischemic optic neuropathy, diabetic retinopathy and multiple sclerosis. Inhibition of caspases through genetic or pharmacological approaches can arrest the apoptotic cascade and protect a proportion of RGC. Novel findings have also highlighted a pyroptotic role of inflammatory caspases in RGC death. In this review, we discuss the molecular signalling mechanisms of apoptotic and inflammatory caspase responses in RGC specifically, their involvement in RGC degeneration and explore their potential as therapeutic targets.

## Bullet points

- Caspase-mediated cell death can occur in normal physiology and pathology.
- Retinal ganglion cells undergo caspase-mediated apoptosis.
- Pyroptosis, a specialised form of inflammatory programmed cell death, mediated by inflammatory caspases, can occur in retinal ganglion cells.
- Inhibition of caspases with pharmacological or genetic inhibitors promotes retinal ganglion cell survival.

## Introduction

Retinal ganglion cells (RGCs) in the ganglion cell layer (GCL) of the inner retina form axons of the optic nerve (ON), which partially decussate at the optic chiasm, project in the optic tract and synapse in the lateral geniculate nucleus (LGN) as well as the superior colliculus, pretectal nucleus and hypothalamus. Optic radiations relay visual information from the LGN to the visual cortex.^1^ The neural retina is an outgrowth of the central nervous system (CNS); consequently after injury, there is limited endogenous axon regeneration and lost RGCs are not replaced, leading to irreversible visual loss.

Caspases, a family of cysteine aspartate proteases, have roles in neuronal pruning during development, inducing RGC death (through apoptosis and pyroptosis) after trauma and disease and promoting RGC axon regeneration. Such processes are attenuated by endogenous and pharmacological inhibitors as well as gene knockdown using short interfering RNA (siRNA) to both understand signalling mechanisms and develop therapeutics to prevent RGC death and promote axon regeneration.

Here we review caspases in apoptotic and pyroptotic RGC death, the novel role of caspases in RGC axon regeneration and the neuroprotective success of caspase-targeting interventions.

## Caspases

Caspases are cysteine aspartate proteases that can be divided into two major phylogenic subfamilies, either interleukin (IL)-1*β*-converting enzyme (inflammatory) or mammalian counterparts of CED-3 (apoptotic) caspases.^2,3^ Caspases are the main components of the apoptotic signalling cascade, although they do also have other non-apoptotic roles, including inflammation.^4,5^ Caspases are activated by proximity-induced dimerisation, within protein complexes, feedback loops and pro-enzyme cleavage.^6,7^

### Apoptotic caspases

Caspases induce apoptosis through initiator and executioner family members: initiator caspases (caspase-2, -8, -9 and -10) activate executioner caspases (caspase-3, -6 and -7) through catalytic cleavage of their activation domain.^5,8^ Activated executioner caspases then hydrolyse or cleave proteins leading to cellular apoptosis.^2^

**Figure 1:**
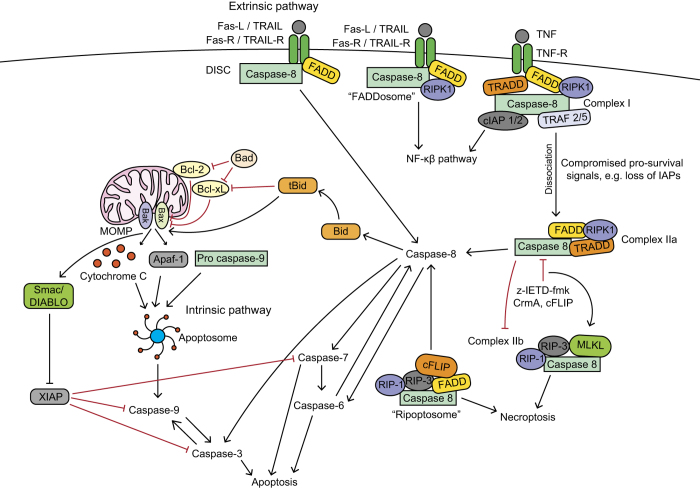
Apoptotic caspases in the canonical intrinsic and extrinsic pathways. Death receptor activation mediates the extrinsic pathway. Fas-R and TRAIL-R recruit FADD^9,10^ and pro-caspase-8,^11^ forming the DISC,^9,12^ leading to proximity-induced caspase-8 activation^11,12^ and downstream activation of executioner caspase-3, -6 and -7.^5^ Caspase-8 can also activate the intrinsic pathway through truncating BH3-interacting domain death agonist (Bid) into tBid, which then promotes Bak and Bax mitochondrial membrane insertion, increasing MOMP and releasing apoptogenic factors,^13^ including Apaf-1, Cytochrome *C* and second mitochondria-derived activator of caspase/direct inhibitor of apoptosis-binding protein with low pI (Smac/DIABLO).^14,15^ Cytochrome C, Apaf-1 and pro-caspase-9 form the septameric apoptosome complex,^16,17^ which activates caspase-9 and successively downstream executioner caspases. Smac/DIABLO indirectly promotes apoptosis by opposing XIAP inhibition of caspase-3, -7 and -9.^22^ Caspase-8 can also form complex I at the TNF receptor, which upregulates the NF-*κ*B survival inflammatory pathway; however, if survival signals are compromised (for example, IAPs) then complex I dissociates from the receptor forming complex IIa, which initiates caspase-8-dependent apoptosis.^19^ Caspase-8 inhibits complex IIb formation and necroptosis and caspase-8 inhibition (for example, through z-IETD-fmk) induces complex IIb formation, causing necroptosis.^20^ The ‘ripoptosome’ complex forms after cellular IAPs (cIAPs) or XIAP inhibition, causing caspase-8-dependent apoptosis and necroptosis.^23,24^

Caspases can be activated through the canonical intrinsic or extrinsic apoptotic pathways (Figure 1). The extrinsic pathway is activated through ligand-activation of tumour necrosis factor (TNF) receptor members^9^ including Fas/CD95 receptor, successive recruitment of adaptor proteins, such as Fas-associated protein with death domain (FADD)^9,10^ and subsequently pro-caspase-8.^11^ Interactions between Fas/CD95, FADD and caspase-8 form the death-induced signalling complex (DISC)^9,12^ and initiate caspase-8 activation,^11,12^ which sequentially cleaves and activates executioner caspase-3, -6 and -7.^5^ Additionally, caspase-8 can cleave the B-cell lymphoma (Bcl)-2 protein family member BH3 interacting domain death agonist (Bid) into truncated Bid (tBid), which stimulates mitochondrial outer membrane permeabilisation (MOMP), releasing apoptogenic factors,^13^ including Cytochrome *C*, apoptotic protease activating factor 1 (Apaf-1), second mitochondria-derived activator of caspase/direct inhibitor of apoptosis-binding protein with low pI (Smac/DIABLO), high-temperature requirement (Htr) A2 (also known as Omi), endonuclease-G and apoptosis-inducing factor.^14,15^

The intrinsic pathway is mitochondria-dependent and activated by intracellular insults, including DNA damage and loss of extracellular membrane integrity, causing MOMP.^13^ Mitochondrial-derived Cytochrome *C* complexes with Apaf-1, recruits and activates pro-caspase-9 in a protein complex termed the apoptosome,^16,17^ allowing successive activation of downstream executioner caspases.^16^ TNF cell surface death receptors and different intracellular complexes also mediate cell death (Figure 1). After TNF-R stimulation, receptor interacting protein kinase (RIPK) 1, TNF-R1-associated death domain protein (TRADD), TNF-R associated factor (TRAF 2/5) and cellular inhibitor of apoptosis (cIAP 1/2) are recruited and form membrane-associated complex I.^18^ TNF-R primarily drives inflammatory gene transcription through the nuclear factor kappa-light-chain-enhancer of B cells (NF-*κ*B) pathway. Reduced pro-survival signals at the TNF-R (for example, loss of IAPs), dissociates complex I causing RIPK1, TRADD, FADD and caspase-8 to form complex IIa, which initiates apoptosis by caspase-8 auto-activation.^19^ Caspase-8 also represses necroptosis (regulated necrosis; mediated by RIPK1 and RIPK3), thus, if caspase-8 is compromised or inhibited, for example, through mammalian inhibitors (CrmA and cFLIPs), pharmacological inhibition (e.g., z-VAD-fmk or z-IETD-fmk) or gene loss, then necroptosis ensues.^20^ Necroptosis activation requires RIPK1, RIPK3 and mixed lineage kinase domain-like protein (MLKL), which form complex IIb.^21^ X-linked IAP (XIAP) directly inhibits caspase-3, -7 and -9^22^ and inhibition of cIAPs and XIAP causes complex II (the 'ripoptosome'; (RIPK1-RIPK3-FADD-caspase-8-cFLIP),^23,24^ which drives caspase-8-mediated apoptosis or caspase-independent necroptosis without the need for receptor ligation.

Caspase-8 also acts as a non-enzymatic scaffold in the assembly of a pro-inflammatory 'FADDosome' (caspase-8-FADD-RIPK1) complex, inducing NF-*κ*B-dependent inflammation.^25^

**Figure 2:**
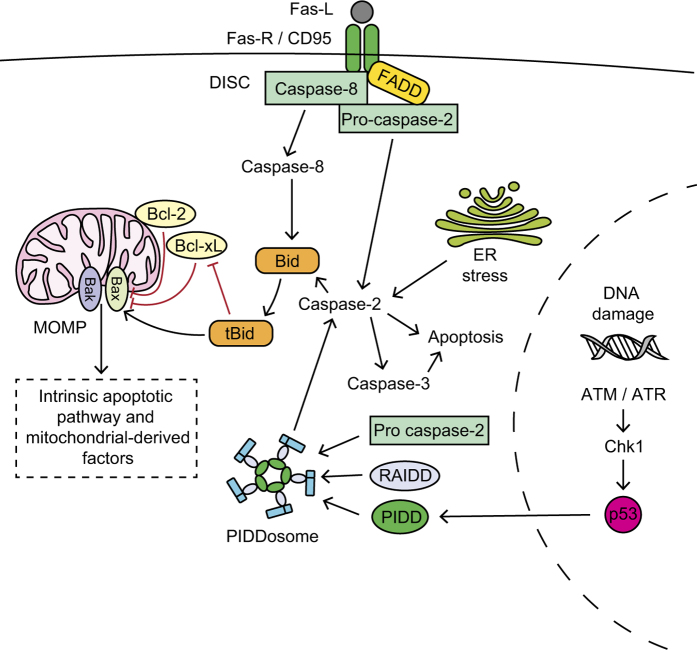
Activation mechanisms of caspase-2. Caspase-2 is activated through DNA damage, upregulation of p53 and formation of the PIDDosome protein complex, which includes p53-induced protein with death domain (PIDD), RIP-associated ICH-1 homologous protein with death domain (RAIDD) and pro-caspase-2.^30,33–35^ Caspase-2 is also activated by endoplasmic reticulum (ER) stress and at the Fas-R within the DISC, alongside Fas-associated protein with death domain (FADD) and caspase-8.^28–32^ Active caspase-2 cleaves and activates caspase-3, cleaves BH3 interacting domain death agonist (Bid; which initiates MOMP and the intrinsic apoptotic pathway) or initiates apoptosis directly.

Uniquely, caspase-2 can act as both an initiator and an executioner caspase, depending on the apoptotic stimuli and does not fit into either the classically described intrinsic or extrinsic apoptotic pathways (Figure 2)^26,27^; its structure resembles that of an initiator caspase due to its caspase recruitment domain but can act as an executioner caspase in response to multiple triggers, including DNA damage, heat shock, endoplasmic reticulum and oxidative stress.^28–32^ DNA damage induces PIDDosome formation: a protein complex that consists of adaptor protein RIP-associated ICH-1 homologous protein with a death domain (RAIDD)^33^ and p53-induced protein with a death domain (PIDD),^30,34,35^ which recruit and activate pro-caspase-2. Caspase-2 can also be activated at the DISC. Caspase-2 can also mediate apoptosis directly from the mitochondrial compartment.^36^

### Inflammatory caspases

**Figure 3:**
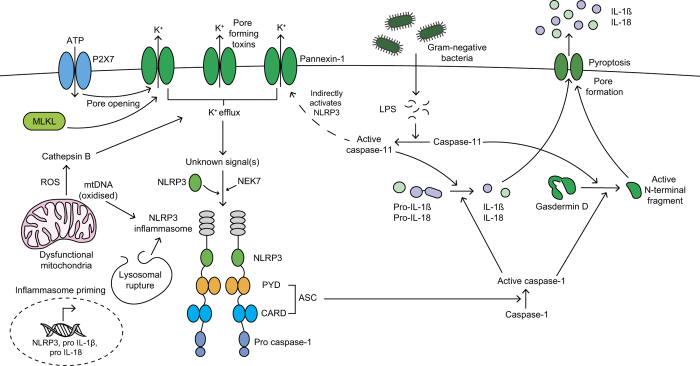
Inflammatory caspase-1 is activated within the inflammasome protein complex;^4,37,38^ which typically consists of a Nod-like receptor (NLR; such as Nod-like-receptor pyrin domain containing 3 (NLRP3)), adaptor protein apoptosis-associated speck-like protein containing a CARD (ASC) and caspase-1.^37–39^ Initial inflammasome priming is required for transcriptional upregulation of inflammasome machinery, such as NLRP3, pro-IL-1*β* and pro-IL-18.^37,38^ A second signal then induces inflammasome assembly and activation. The NLRP3 inflammasome is activated by lysosomal rupture, reactive oxygen species (ROS), oxidised mitochondrial DNA (mtDNA) and cathepsin B.^38,43^ Potassium (K^+^) efflux is a common NLRP3-activation mechanism, induced by P2X7-mediated pore opening, pore-forming toxins, pannexin-1 or MLKL-mediated pore opening.^44^ The NLRP3 inflammasome activates caspase-1, which cleaves precursor cytokines IL-1*β* and IL-18 into their active forms and gasdermin-D into its N-terminal fragment. The N-terminal fragment of gasdermin-D forms a plasma membrane pore facilitating pro-inflammatory cytokines release and inducing pyroptosis.^40–42^ Gram-negative bacterial lipopolysaccharide (LPS) can activate caspase-11,^40^ which also cleaves gasdermin-D cleavage and indirectly activates the NLRP3 inflammasome via pannexin-1.^47^

Inflammatory caspases (-1 or -11 in mice and -1, -4 and -5 in humans) can be activated in the inflammasome protein signalling complex (Figure 3).^4,37,38^ Inflammasomes are large multimeric protein complexes that sense pathogen- and host-derived danger signals and typically comprise of a Nod-like receptor (NLR), adaptor protein apoptosis-associated speck-like protein containing a CARD (ASC) and caspase-1.^37–39^ The main functions of the inflammasome are to activate caspase-1 to cleave precursor cytokines IL-1*β* and IL-18 into their mature active forms and induce pyroptosis (a lytic form of cell death). Active caspase-1 also cleaves gasdermin-D into its cytotoxic N-terminal fragment, which forms a plasma membrane pore, releasing pro-inflammatory cytokines.^40–42^ Inflammasome activation is a two-step process: initial inflammasome priming is required for transcriptional upregulation of machinery including Nod-like-receptor pyrin domain containing 3 (NLRP3) and pro-IL-1*β*,^37,38^ followed by the trigger, such as a pathogen-associated molecular pattern (PAMP) or a damage-associated molecular pattern (DAMP), which induces inflammasome assembly and activation.

The canonical NLRP3 inflammasome can be activated by PAMPs (for example, *Staphylococcus aureus*) and host-derived DAMPs (e.g., ATP, phagolysomal rupture, cathepsins release, ion flux, calcium influx, mitochondrial reactive oxygen species and oxidised mitochondrial DNA).^38,43^ Potassium efflux has been proposed as a universal trigger for NLRP3 activation,^44^ including P2X7 receptor-mediated potassium pore opening, pannexin-1 and pore-forming toxins.^44^ However, potassium efflux is not a common mechanism for all activation pathways.^45,46^

Caspase-11, -4 and -5 can be activated by bacterial lipopolysaccharide-induced oligomerisation,^40^ cleaving gasdermin-D and indirectly activating the NLRP3 inflammasome via pannexin-1 and potassium efflux.^47^ NLRP3 inflammasome can also be activated by caspase-8 – which also directly cleaves IL-1*β*.^48,49^ MLKL translocates to the cell membrane and disrupts it, triggering potassium efflux and assembly of the NLRP3 inflammasome.^50^ MLKL activation also provides a mechanism for processing and release of IL-1*β* independently of gasdermin-D.^50^

## Anticaspase treatments: pharmacological, gene knockdown and siRNA techniques

A number of specific and broad-spectrum caspase inhibitors are based upon the amino-acid sequence of caspase substrate cleavage sites, acting as pseudoenzymes for active caspases and therefore competitive inhibitors. Broad-spectrum inhibitors include Boc-D-fmk, Q-VD-Oph (inhibits caspase-1, -2, -3, -6, -8 and -9), z-VAD-fmk (inhibits all caspases but caspase-2 very weakly).^51–54^ Specific caspase substrate cleavage sites include WEHD (caspase-1), YVAD (caspase-1), VDVAD (caspase-2), DEVD (caspase-3), LEVD (caspase-4), VEID (caspase-6), LETD (caspase-6), IETD (caspase-8 and -10) and LEHD (caspase-9)^53,55,56^.^2,3^ Caspase peptide inhibitors are linked to chemical groups that improve permeability, efficacy and stability of the compound. Peptides linked to aldehydes (or nitriles or ketones) are reversible inhibitors (e.g., Ac-DEVD-CHO) and bind to the catalytic site but do not irreversibly chemically alter the enzyme, whereas peptides linked to halmethylketones (chloro or fluoro) (e.g., z-VAD-fmk) bind irreversibly. The chemical group -fmk is non-specific.^56,57^

Cross-reactivity with 'off-target' caspases limits interpretation of many studies using these inhibitors. The sequence DEVD (caspase-3) also binds to caspase-6, -7, -8 -9 and -10, similarly VDVAD (caspase-2) binds caspase-3 and -7 and LETD (caspase-6) binds caspase-3, -8 and -9.^55,58,59^ VEID has a stronger efficacy for caspase-3 than its target caspase-6, IETD has a stronger efficacy for caspase-3 and -6 than its target caspases -8 and -10 and LEHD has a stronger efficacy for caspase-8 and -10 than their intended substrate IETD, and LEHD also binds caspase-3 and -6.^55,58,59^ In addition, z-VAD-fmk also binds other cysteine proteases, such as calpains and cathepsins.^51^

Caspase activity can also be modulated by siRNA-mediated gene knockdown, dominant-negative proteins and conditional and global gene knockout. RNA interference technology may cause alternative signalling induced by short RNA species and off-target effects, thus appropriate controls are still critical.^60^

## Caspases and RGC death

Caspase-dependent RGC death occurs after eye and brain injuries, in retinal and optic nerve degenerative disorders^61,62^ and during development.^63,64^ Common mechanisms of degeneration between different conditions could lead to broadly translatable therapeutics. Caspase involvement in RGC death in animal models, primary cell culture and human postmortem specimens are highlighted in this section. Relative efficacy of neuroprotection is shown for direct caspase inhibitors in Table 1 and upstream indirect inhibitors in Table 2.

### Endogenous caspase activity and inhibition in RGC

#### Development

Caspase-dependent apoptosis is important in pruning neuronal, including RGC, numbers after normal developmental overproduction,^63,65^ causing an ~50% reduction in RGC numbers shortly after cell birth, which can be prevented by broad-spectrum caspase inhibitor, Boc-D-fmk.^66,67^ Caspase-3 is pivotal in neuronal developmental apoptosis, with active caspase-3 co-localising to terminal deoxynucleotidyl transferase dUTP nick end labelling (TUNEL)-positive RGC in 2–6-day chick embryos,^67^ and caspase-3 inhibition, using z-DEVD-fmk, reducing TUNEL-positive cells by ~50% and increasing RGC numbers, axons and GCL thickness.^67^ Moreover, BARHL2, a member of the *Barh* gene family, which suppresses caspase-3 activation, is essential for developmental preservation of normal complement of RGC subtypes.^68^

Supporting this, caspase-3 knockout mice express a brain-specific phenotype with excessive neuronal numbers and cellular disorganisation, dying at 1–3 weeks of age.^3,69^ Similarly, caspase-9 knockout results in a selective CNS phenotype, characterised by severe brain malformations and high perinatal lethality without gross abnormality of other body parts.^70,71^ Caspase-2 (NEDD2) gene expression is elevated during neurogenesis and downregulated in the mature brain and retina.^72,73^ However, caspase-2 knockout mice develop normally and lack overt phenotypic abnormalities, with minimal CNS or retinal defects. The role of caspase-2 in RGC neurogenesis is therefore unclear. In more mature mouse retinae, there are no alterations in caspase-3, -6, -7, -8 or -9 expression between 6 and 24 weeks.^74^ However, there was a reduction in cIAP-1 suggesting a possible role for caspases at this stage.^74^

### Induced caspase activity and anti-caspase treatment in RGC

#### Optic neuritis

Multiple sclerosis (MS) is an autoimmune, demyelinating CNS disease and a major cause of non-traumatic disability in young adults. Optic neuritis involves ON inflammation and demyelination and is a common presenting feature of MS^75^ associated with visual loss. The extent of visual recovery after acute optic neuritis is influenced by demyelination, axonal loss and RGC death.^76^ The experimental autoimmune encephalomyelitis (EAE) model is the most common MS animal model induced by myelin oligodendrocyte glycoprotein (MOG) peptide administration causing autoimmunity, inflammation and neurodegeneration.^77,78^ In the EAE rat model cleaved caspase-3 immunolocalised to Fluoro-Gold-labelled RGC suggesting that RGC die by apoptosis,^77^ though in the EAE mouse model only full-length caspase-3 immunostaining is present in the GCL.^78^ RGC NADH dehydrogenase (mitochondrial electron transport chain) overexpression suppresses RGC death, rescuing 88% of RGC and reducing cleaved caspase-3 immunostaining in Thy1-labelled RGC.^79^ Treatment with erythropoietin (EPO) reduces RGC death and active caspase-3 levels, supporting a critical role for caspase-3.^80^ Various regulators upstream of caspase-3 are also neuroprotective (Table 2).

In a refined mouse model of MS, the MOGTCR×Thy1CFP mouse, which develops optic neuritis only, either spontaneously or following induction with Bordetella pertussis toxin,^81^ RGC express active caspase-2 and intravitreal injection of a modified siRNA against caspase-2 (siCASP2) protects ~80% of RGC against apoptosis and axonal degeneration,^81^ suggesting a critical role for caspase-2 in RGC apoptosis after optic neuritis.

#### Traumatic optic neuropathy

Traumatic optic neuropathy (TON) is a major cause of visual loss after brain and eye injury. TON can be either direct – when the ON is crushed or severed – or more commonly indirect, when brain or ocular injury causes secondary RGC death or ON injury. Spontaneous recovery occurs in a minority of patients.^82^ However, the most common outcome is permanent blindness, and at present, there is no treatment that improves outcome.^83,84^ Direct TON can be caused by penetrating injury, such as craniofacial fractures, or direct compression from orbital haemorrhage.^85^ ON transection (ONT) and ON crush (ONC) in animal models can be used to study degenerative mechanisms and evaluate neuroprotective and regenerative therapies.^86,87^

RGC death after ON injury is progressive and the severity is dependent upon type of lesion and distance from the eye.^88,89^ After direct TON, RGC begin to degenerate 5 days after axotomy,^90^ and 90% die between 7 and 14 days^86,89,91,92^ through caspase-dependent apoptosis.^93,94^ Cleaved caspase-2,^91,95,96^ -8,^61,97^ -9,^90,98,99^ -3,^90,100–105^ -6^61^ and -7,^102,106^ as well as inflammatory caspases -11^107^ and -1,^108^ have all been detected in RGC after crush or axotomy, highlighting the crucial role played by caspases in axotomy-induced RGC death.

Caspase-3 is activated after RGC axotomy,^90,100–105^ and z-DEVD-fmk inhibition reduces RGC death.^99,101–103,109, 110^ However, z-DEVD-fmk also inhibits caspase-6, -7, -8 -9 and -10^55,59^ and neither delayed nor multiple treatments of z-DEVD-fmk improved the RGC survival.^101^ Caspase-3 is also indirectly reduced in RGC-neuroprotective therapies, such as either Rho-associated protein kinase (ROCK) inhibition^111,112^ or treatment with the broad-spectrum histone deacetylase inhibitor, valproic acid.^113,114^ Moreover, a rabbit fluid percussion injury model of indirect TON increases cleaved caspase-3 in retinal lysate, where full-length caspase-3 is localised to RGC and pharmacological inhibition with z-DEVD-fmk is RGC neuroprotective.^115^

Caspase-7 gene knockout also protects a limited proportion of RGC after axotomy^106^ and pharmacological inhibition of caspase-6 and -8, using z-VEID-fmk and z-IETD-fmk or a dominant-negative against caspase-6 (CASP6 DN) provides some RGC neuroprotection and promotes regeneration.^61^ Although caspase-6 is localised to RGC and some microglia, regeneration is an indirect effect of ciliary neurotrophic factor (CNTF) production by retinal glia.^96^ In addition, combined caspase-8 and -9 inhibition provides additive survival benefits compared with single inhibition,^90,97,102^ which may suggest either that both intrinsic and extrinsic apoptotic pathways are activated following direct optic nerve injury or that there are increased off-target effects. Inhibition of caspase-8 can also promote caspase-independent RGC death, such as necroptosis.^20^

Recent studies have indicated a pivotal role of caspase-2 in apoptotic RGC injury.^91,95,96,116,117^ After ON axotomy and crush, active caspase-2 is exclusively localised to RGC, and its inhibition using siRNA provides significant neuroprotection.^91,95,96^ For example, intravitreal administration of either siCASP2^91^ or the pharmacological inhibitor z-VDVAD-fmk^95^ protect 98% and 60% of RGC, respectively, for up to 30 days and >95% of RGC are protected from death for 12 weeks if siCASP2 is injected every 8 days.^116^ Pharmacological inhibition with z-VDVAD-fmk also inhibits caspase-3 and -7,^59^ though activation of these caspases was not affected. The siCASP2 is being developed by Quark Pharmaceuticals Inc. and is currently in Phase III clinical trials for ischaemic optic neuropathy and glaucoma.^116^

NLRP3-induced neuroinflammation promotes RGC death after partial ONC.^108^ NLRP3 expression is upregulated in retinal microglia and NLRP3 inflammasome activation upregulates retinal cleaved caspase-1 and IL-1*β*, which is prevented in NLRP3 knockout mice, in which RGC are protected against axotomy-induced RGC death.^108^ The P2X7 ionotropic ATP-gated receptors are implicated in RGC degeneration; P2X7-mediated potassium efflux induces NLRP3 inflammasome formation and caspase-1 activation.^44^ P2X7 receptor-deficient mice displayed delayed RGC loss and reduced phagocytic microglia at early time points after RGC axotomy.^118^ Intravitreal administration of a selective PX27 receptor antagonist A438079 delayed RGC death, suggesting P2X7 receptor antagonism as a potential therapeutic strategy.^118^ Caspase-11 expression is also upregulated in RGC after ONC and ONT.^107^

#### Primary ocular blast injury

Although direct ON injury results in rapid RGC degeneration, indirect blast-induced TON is delayed and progressive. After explosive blast, the sonic blast-wave causes primary blast injury (PBI), which can cause indirect TON.^119,120^ Secondary blast injury causes direct and indirect TON, when explosively propelled fragments impact the eye, head and ON. Blast injury represents a significant threat to military personnel in modern warfare causing visual loss.^121,122^ Multiple studies have demonstrated increased cleaved caspase-3 in the GCL and ON between 3 and 72 h after whole animal^123,124^ and direct local ocular blast exposures.^125^ Moreover, caspase-3 activation displays a cumulative effect after multiple exposures,^124^ which is comparable to repeated exposure in combat, potentially leading to worse structural and functional visual outcomes.^126^ Additionally, an alternative model using trinitrotoluene (TNT) explosives detected active caspase-3 exclusively in photoreceptors and not RGC.^127^ Other apoptotic markers, such as Bax, Bcl-xL and Cytochrome *C* are also elevated in the retina up to 24 h after blast injury.^125^ DBA/2J mice lack ocular regulatory mechanism of immune privilege in the anterior chamber,^128^ and are thus used as a closed globe injury model to approximate features of open globe injury, without complications of infection.^129^ In this model, full-length inflammatory caspase-1 is immunolocalised to the inner nuclear layer (INL) and GCL in control retinae, but immunostaining declines after blast injury,^129^ suggesting caspase-1 cleavage. However, necroptotic markers RIPK1 and RIPK3 have increased retinal expression, with RIPK1 localised to outer nuclear layer (ONL), INL and Müller glia and RIPK3 in the ONL, INL and GCL 3 and 28 days post-ocular PBI.^130^ These findings suggest potential activation of necroptotic or pyroptotic death pathways.

Although caspase activation immediately follows blast injury, RGC death does not occur until later time points,^130^ with retinal nerve fibre layer (RNFL) thickness unchanged for 3 months postblast.^131,132^ Axonal degeneration at 28 days after ON demyelination^130^ suggests that, as in direct TON, ON degeneration may precede RGC death.^133^ Research into blast-induced RGC degeneration is in its infancy. However, roles for apoptotic and potentially inflammatory caspases in RGC death are apparent.

#### Excitotoxicity-induced RGC death

Excitatory neurotransmitter glutamate is linked to retinal degeneration, for example, in glaucoma, through overactivation of *N*-methyl-d-aspartate (NMDA) receptors, calcium overload and subsequent mitochondrial dysfunction. Excitotoxicity-induced RGC death is caspase dependent; broad-spectrum caspase inhibition preserves GCL cells.^134^ Intravitreal caspase-3, -6, -8 and -9 inhibitors, DEVD-fmk, VEID-fmk, IETD-fmk and LEHD-fmk respectively, significantly protect RGC, but caspase-1 and -4 inhibition, using YVAD-fmk, does not,^135^ suggesting that excitotoxicity-induced RGC death is apoptotic but not pyroptotic. The greatest RGC neuroprotection is provided by DEVD-fmk, which inhibits caspase-3 and also -2, -6, -7, -8, -9 and -10. The latter, LEHD-fmk (intended for caspase-9), is most specific for caspase-3 and -8 and also inhibits -6 and -10.^58,59,135^

The IQACRG amino-acid sequence is conserved in the active site of caspase-1, -2, -3, -6 and -7 and the synthetic peptide, with amino-acid sequence IQACRG, acts as an enzymatically inactive caspase mimetic, thus binds to caspase substrates as a pseudo-enzyme and protects them from proteolysis by caspases. Treatment with IQACRG caspase mimetic protects RGC from excitoxicity-induced death both *in vivo* and in primary culture.^136^

#### Light-induced retinopathy

Light exposure can cause light-induced retinal damage (LIRD) and blindness,^137,138^ and a light-toxicity animal model induces photoreceptor and caspase-dependent RGC apoptosis.^139^ Cleaved caspase-3 is elevated in RGC 6 h after toxic light exposure and reaches a peak after 3 days,^140–142^ co-localising with increased staining for Ras homologue enriched in the brain (RHEB), cyclic AMP response element modulator-1 (CREM-1), transcription initiator factor IIB (TFIIB), pyruvate kinase isozyme type M2 (PKM2), SYF2 pre-mRNA splicing factor (SYF2) and RNA-binding motif protein, X-linked (RBMX), which are all involved in cell death pathways.^140–145^ Nuclear factor of activated T cells, cytoplasmic 4 (NFATc4) (a component of T-cell activation and a regulator of the immune response) are also co-localised with cleaved caspase-3, caspase-8 and Fas-L in RGC, suggesting that NFATc4 may upregulate Fas-L and participate in RGC apoptosis.^146^ Intravitreal mitogen-activated protein kinases/extracellular signal-regulated kinases (MAPK/ERK) inhibitor reduces PKM2 and active caspase-3 protein expression, suggesting that light-induced RGC apoptosis is in part dependent on MAPK/ERK pathway.^141^ Together, these studies show that RGC apoptosis is correlated with caspase-3 cleavage but not that RGC death in LIRD is caspase-3 dependent.

#### Ischaemic RGC death

Retinal ischaemia is a common cause of visual impairment and sight loss^147^ and can be experimentally induced by clamping or ligation of the ophthalmic artery, raising intraocular pressure (IOP) or bilateral common carotid artery occlusion.^148–151^ The degree of RGC loss after ischaemic injury is dependent upon the length of ischaemic interval and is progressive. For example, after 45 min of ligation, ischaemia induces ~50% of RGC to degenerate over a 2-week period, whereas 120 min induces death of 99% over 3 months.^151^

Ischaemic RGC degeneration is caspase dependent, evidenced by neuroprotection with broad-spectrum caspase inhibitors (Q-VD-OPH and Boc-aspartyl-fmk).^62^ In Thy1-positive RGC, full-length caspase-2 expression is increased 1,^152^ 6,^153,154^ 24^152,154^ and 72 h^152^ after ischaemia and antisense oligonucleotide inhibitor of caspase-2 (antisense Nedd-2 oligonucleotide 5′-QGCTCGGCGCCGCCATTTCCAGL-3′) protected inner retinal thickness at 7 days.^152^ Brain-derived neurotrophic factor (BDNF) is also RGC neuroprotective and reduced caspase-2 expression.^153^ Full-length caspase-3 immunolocalised to the GCL 4 h after injury^155^ and preinjury intravitreal siRNA caspase-3 injection was RGC neuroprotective,^156^ though other studies have found full-length caspase-3 to be exclusively in the INL and ONL.^152^ Valproic acid, a broad-spectrum histone deacetylase inhibitor, protects RGC after ischaemic reperfusion (I/R) injury caused by raised IOP,^113,114,157^ reducing cleaved caspase-3 and -12 expression.^114,157^

Pannexin-1 is a mammalian cell membrane channel-forming protein that acts as a diffusional pathway for ions and small molecules. Pannexin-1 facilitates neurotoxicity in the ischaemic brain and retinal pannexin-1 gene knockout suppresses inflammasome-mediated caspase-1 activation and IL-1*β* production 3 h after ischaemic injury and reduces RGC degeneration at 14 days.^158^ Administration of YVAD-fmk (caspase-1, -4 and -5) protects inner retinal morphology in some, but not all, studies,^152,154,155^ leaving the role of caspase-1 in question. P2X receptor stimulation induces ATP influx, potassium ion efflux and downstream NLRP3 inflammasome and caspase-1 activation.^37,38^ During stimulated ischaemia (oxygen/glucose deprivation) of human organotypic retinal cultures, P2X receptor stimulation causes RGC death, suggesting possible involvement of NLRP3 inflammasome and caspase-1.^159^

RGC axon degeneration after central retinal artery occlusion is mediated by the mitochondrial intrinsic apoptotic pathway^160^ – cytosolic Bax, a pro-apoptotic Bcl-2 family member, levels are decreased at 3 and 6 h post injury, whereas mitochondrial Bax levels are elevated at 3, 6 and 24 h, suggesting that Bax translocates to the mitochondria.^160^ In addition, cytosolic Cytochrome *C* levels are elevated at 3 h post injury but not at 6 and 24 h, and cleaved caspase-9 levels are elevated at 3 h.^160^

RGC are protected by intravitreal caspase-6 and -8 inhibitors (z-VEID-fmk and z-IETD-fmk) and siRNA against caspase-6 and -8 (siCASP6 and siCASP8) after I/R injury.^161^ Two different siRNA were used for each caspase making off-target effects unlikely. Caspase-6 inhibition may act indirectly by increasing retinal glial CNTF production.^96^ Two weeks after ischaemia, z-VEID-fmk (caspase-6, but also -3 and -7) and z-IETD-fmk (caspase-8 but also -3, -6, and -10) protect only a small proportion of RGC, whereas both siCASP8 and siCASP6 administration elevate RGC survival by ~ 60%.^161^ This suggests that small peptide inhibitors are less effective, as they act as a competitive inhibitor for the caspase substrates, whereas siRNA gene knockdown reduces caspase gene expression and could affect non-apoptotic caspase roles, such as caspase-8 in complex IIb, 'FADDosome', 'ripoptosome' and inflammasome formation.^20^

#### Glaucoma

Glaucoma is a complex, multifactorial disease affecting >60 million people worldwide^162^ and is associated with raised IOP causing RGC death. Genetic background^163^ and age^164^ are also associated with disease development. Glaucoma is currently treated by IOP control; however, there is an unmet clinical need for a neuroprotective treatment.

Acute severe IOP elevation induces I/R injury, but models use less severe IOP elevation to simulate glaucoma, include the photocoagulation laser model,^165^ injection of hypertonic saline solution,^166^ injection of paramagnetic microspheres into the anterior chamber, suture-pulley compression,^167^ intracameral transforming growth factor beta (TGF-*β*) injection^168^ and AAV-TGF-*β* transfection to induce trabecular meshwork fibrosis.^169^

Apoptotic caspases -3, -8 and -9 are cleaved in RGC after a period of elevated IOP^166,167,170–176^ and inflammatory caspases -1, -4 and -12 are also upregulated.^170^

In response to acute elevated IOP, NLRP3 inflammasome and IL-1*β* production are induced,^177,178^ mediated through high-mobility group box-1 (HMGB1) via the NF-*κ*B pathway.^178^ HMGB1 promotes NLRP3 and ASC elevation leading to caspase-1 maturation. Caspase-8 acts upstream of the NF-*κ*B HMGB1-caspase-8 pathway and induces the activation of NLRP3 and IL-1*β* production.^178^ Toll-like receptor 4 (TLR4) activation increases macrophage caspase-8 expression upregulating IL-1*β* though the NF-*κ*B pathway^178^ and causes RGC death through the extrinsic pathway. Caspase-8 inhibition, using intravitreal z-IETD-fmk, reduces RGC death through NLRP1 and NLRP3 downregulation, though inhibition of a direct effect of caspase-8 (or other caspases) inhibition on the extrinsic apoptotic pathway is not excluded. Caspase-8 inhibition completely suppresses retinal IL-1*β* expression, but caspase-1 inhibition, using z-YVAD-fmk, does not, suggesting that caspase-8 regulates IL-1*β* expression through caspase-1-dependent and -independent pathways.^177^

Primary open-angle and normal-tension glaucoma patients display serum autoantibodies against retinal and ON antigens.^179–182^ A 'glaucoma-like' syndrome, without direct damage to the retina or ON, has been induced using immunisation of ON homogenate causing RGC degeneration,^179,183^ with increased GCL full-length caspase-3 expression at 14 and 22 days after immunisation.^179^ However, RGC numbers did not decline until 22 days after immunisation.^179^

#### Diabetic retinopathy

RGC degenerate early in the disease process in the human diabetic retinopathy (DR) retinae demonstrated by scanning laser polimetry showing reduced RNFL thickness in DR patients compared with healthy controls.^184–186^ TUNEL-positive RGC are increased in diabetic rats and in human postmortem retinae^187^ and cleaved caspase-3, caspase-9, Fas and Bax localise to RGC.^188,189^

Diabetes mellitus develops in the Akita, insulin gene mutation (Ins2) mouse, after streptozotocin (STZ; toxic to *β* cells) administration, and in the Otsuka Long-Evans Tokushima fatty rats (OLETF; develop insulin resistance).^190–193^ In STZ diabetic mice, retinal caspase activity (assessed with a variety of non-specific substrates) is increased 8 weeks after induction and GCL counts are reduced by 20–25% 14 weeks after induction, with TUNEL positivity and cleaved caspase-3 in the GCL, suggesting RGC apoptosis.^192,194^ Caspase-2, -8 and -9 activity (using substrate sequences VDVAD, IETD and LEHD) transiently increases initially. By 4 months, caspase-3 activity increases and caspase-1, -3, -4 and -5 activities remain elevated,^194^ corroborated by elevated cleaved caspase-8 and -3 levels in whole retinal lysates^195^ and caspase-3 GCL immunolocalisation.^196^ In primary retinal explants exposed to high glucose media, there are more cleaved caspase-3- and -9-positive RGC compared with explants in normal glucose media.^197^

## Caspases and RGC axon regeneration

In addition to promoting RGC survival, caspases promote RGC axon regeneration after ON injury. Pharmacological inhibition of caspase-6 and -8, using z-VEID-fmk and z-IETD-fmk, provide RGC neuroprotection and promote limited RGC axon regeneration,^61^ with few axons extending >1000 μm beyond the lesion site. Similarly, few RGC axons regenerated through the lesion site with inhibition of caspase-6 by a dominant negative (CASP6 DN)^96^; however, combined suppression of caspase-2 and -6 using siCASP2 and CASP6 DN promoted significant regeneration, with an average of 195±9 axons growing beyond 1000 μm.^96^ Although caspase-6 is localised to RGC and some microglia, the neuroprotective and pro-regenerative effects of caspase-6 inhibition are mediated indirectly by CNTF upregulation in retinal glia and are blocked by suppression of gp130 and the JAK/STAT pathway.^96^ These studies reveal a novel non-apoptotic role for caspases and warrants further investigation.

## Conclusion

Postmitotic CNS neurons, including RGC, do not regenerate their axons after trauma or injury; hence RGC trauma or disease can lead to permanent visual loss. Understanding the signalling pathways in RGC injury is vital for the development of therapeutic interventions, such as pharmacological inhibitors, RNA interference technology or gene therapies. Caspases, a family of cysteine aspartate proteases, mediate RGC death in physiology, such as during development, as well as trauma and disease, and their inhibition can prevent RGC death. Caspase-3 is implicated during RGC developmental pruning, whereas most apoptotic and inflammatory caspases are implicated in trauma and disease, with siRNA knockdown of caspase-2 providing the greatest neuroprotection after axotomy. Non-apoptotic roles of caspases, such as inflammatory pyroptotic death or facilitating formation of necroptotic complexes are also critical in RGC death. Caspases also have a novel role in RGC axon regeneration; in particular, caspase-6 inhibition mediates regeneration indirectly through CNTF upregulation in retinal glia. Understanding the key pathways for caspase-dependant RGC death is fundamental to the development and effective translation of neuroprotective treatments from preclinical studies to clinical practice.

## Figures and Tables

**Table 1:** Treatments directly targeting caspases in RGC degenerative disease

*Caspase*	*Model*	*Inhibitor*	*Time at the end of the study (days)*	*Percentage surviving RGC (% untreated)*	*Percentage surviving RGC (% treated)*	*References*
Broad spectrum	ONT	z-VAD	14	16.8^a^	34.5^a^	^61^
	75 min raised IOP	Q-VD-OPH	7–21	39–64	63–71	^62^
Caspase-1	ONC	NLRP3−/−	3–28	78–13	89–25	^108^
	NMDA-RGC explants	YVAD-fmk	2	18	12	^135^
Caspase-2	ONC	z-VDAD-fmk	15	12.3	60	^95,96^
	ONC	siCASP2	21–84	10–7	95–96	^91,116^
	Optic neuritis	siCASP2	21	65.5	79.3	^81^
Caspase-3	ONT	z-DEVD-cmk	7–28	10–34.3	24.3–47.4	^61,100,101^
	NMDA-RGC explants	DEVD-fmk	2	18	26	^135^
Caspase-3 and -6	NMDA-RGC explants	DQMD-fmk	2	18	41.6	^135^
Caspase-6	ONT	SIMA 13a	13	16.8^a^	37^a^	^61^
	ONC	CASP6 DN	21	14.2	39.4	^96^
	ONT	z-VEID	14	16.8^a^	48.2^a^	^61^
	NMDA-RGC explants	VEID-fmk	2	18	41.6	^135^
	30 min artery ligation	z-VEID-fmk	14	33.9	46.2	^161^
	30 min artery ligation	siCASP6	14	30^a^	48^a^	^161^
Caspase-7	ONT	CASP7−/−	28	38	76	^106^
Caspase-8	ONT	z-IETD (+/−) -fmk	14	16.8^a^	31.5–60.7^a^	^61,97^
	ONT	IETD-CHO	14	NA	33.1	^97^
	NMDA-RGC explants	IETD-fmk	2	18	27	^135^
	30 min artery ligation	z-IETD-fmk	14	33.9	42.2	^161^
	30 min artery ligation	siCASP8	14	30.0^a^	48.4^a^	^161^
Caspase-8 and -9	ONT	z-IETD-fmk and z-LEHD-fmk	14	NA	38.7	^97^
Caspase-9	ONT	z-LEHD- (+/−) fmk	14	16.8^a^	29.1–34.9^a^	^61,97^
	NMDA-RGC explants	LEHD-fmk	2	18	39	^135^

Specific pharmacological inhibitors, gene knockdown (i.e., siRNA) or gene knockout (−/−) treatment are displayed with the percentage of surviving RGC in untreated and treated retinae.

a For calculations, values for uninjured Fluoro-Gold and RBPMS RGC counts not stated in Shabanzadeh *et al*.^161^ and values for identical animals (Sprague Dawley female adult rats) with Fluoro-Gold and RGC counts per mm^2^ were used from Weishaupt *et al*.^97^

**Table 2:** Treatments that affect targets upstream of caspases and prevent RGC death

*Disease*	*Injury*	*Treatment*	*Effect on caspase by treatment*	*End of the study (days)*	*Effect on RGC*	*References*
Direct ON injury	ONC	ROCK inhibition	Reduced cleaved caspase-3 immunostaining in GCL and primary RGC culture lysate	14	ROCK shRNA increases RGC survival to 143% of EGFP shRNA control	^111,112^
	ONC	Calcineurin inhibition	Reduced cleaved caspase-9 protein	—	ND	^98^
	ONC	Deletion of CHOP	Reduced full-length caspase-3 immunostaining	14	CHOP KO mice had 52% surviving RGC compared with 24% in sham	^198^
	ONT	Kv1.3 siRNA	Reduced caspase-3 and -9 mRNA expression	14	KV 1.3-1169 siRNA increases RGC survival 3.5-fold compared with control	^199^
	ONC	Valproic acid (VPA)	Reduced cleaved caspase-3 RGC immunostaining	14	VPA treatment has 44% surviving RGC compared with 27% in vehicle	^113,114^
Glaucoma	Hypertonic saline injections into limbal vein	Morphine	Reduced cleaved caspase-3 and -8 protein	56	Morphine treatment has 65.9% surviving RGC compared with 17.3% in control	^166^
	Laser photocoagulation	Cobra venom factor (CVF; complement depletion)	Reduced cleaved caspase-8 and -9 protein	42	CVF treatment has 41.5% surviving RGC compared with 28.4% in control	^165^
	Suture pulley compression	C-Jun N-terminal kinase (C-JNK) inhibitor	Reduced cleaved caspase-3 immunostaining	0.5	C-JNK inhibition has 23.6% of RGC as TUNEL positive compared with 52.4% in vehicle control and 1.49% in uninjured	^167^
	Saline injection into anterior chamber	Cyclosporine A (CSA; inhibits cyclophilin D and MPTP)	Reduced cleaved caspase-3 protein, immunolocalised to RGC	14	CSA treatment has 93% surviving RGC compared with 77% in ischaemic	^200^
	Translimbal photocoagulation laser model	Minocycline, tetracycline antibiotic	Reduced caspase-1 and -4 but not caspase-8 and -12 gene expression	8	ND	^170,201^
Glutamate excitotoxicity	Glutamate – primary rat RGC culture	Pilocarpine (M1 muscarinic receptor agonist)	Reduced caspase-3 gene expression and full-length protein	1	Cell viability is 42% after 1 mM of glutamate, increases by 32% with pilocarpine treatment	^153,202^
	NMDA administration	Thioredoxin (TRX)	Reduced cleaved caspase-3 and -9 protein	7	TRX treatment has 56.6% surviving RGC compared with 13.4% in control	^203^
Ischaemic injury	Ischaemic reperfusion injury	Brain-derived neurotrophic factor (BDNF)	Reduced full-length caspase-2 GCL immunostaining	7	BDNF treatment has 69.6% surviving RGC compared with 44.1% in sham	^153,154^
	Ischaemic reperfusion injury	VPA	Reduced cleaved caspase-12 protein	7	VPA treatment has 83.5% surviving GCL cells compared with 57.5% in sham	^157^
Branch retinal vein occlusion (BRVO)	Laser photocoagulation	Minocycline, tetracycline antibiotic	Reduced cleaved caspase-3 immunostaining in GCL	7	*In vivo* OCT imaging shows increased RNFL+GCL thickness 3 days after minocycline. Minocycline has 55.2% RGC compared with 46.8% in saline control	^204^
Diabetic retinopathy	STZ	Somatostatin (SST)	Reduced cleaved caspase-8 and -3 protein	14	Reduced TUNEL cells in GCL, 36.8% in STZ compared with 13.7% in treated	^195^
	STZ	Edaravone (free radical scavenger)	Reduced cleaved caspase-3 protein	28	Reduced TUNEL cells in GCL, 42% in vehicle compared with 9.5% in treated	^205^
	High glucose primary RGC culture	Erythropoietin (EPO; antioxidant)	Reduced full-length caspase-3 and -9 protein	—	Reduced apoptotic Hoechst 33358-stained cells, 49.1% in high glucose compared with 25.7% in EPO treated	^206^
	High glucose primary RGC culture	L-Carnitine (endogenous mitochondrial membrane compound)	Reduced full-length caspase-3 and -9 protein	—	Reduced apoptotic Hoechst 33358 stained cells, 49.1% in high glucose compared with 15.7% in L-Carntine treated	^207^
Optic neuritis	EAE model	EPO	Reduced cleaved caspase-3 immunostaining	8	EPO treatment has 55% RGC surviving compared with 30% in vehicle control	^80^
PBI	Blast wave	Compound 49b (beta-adrenergic receptor agonist)	Reduced cleaved caspase-3	3	ND	^125^

Abbreviations: CHOP, CCAAT/enhancer binding homologous protein; EAE, experimental autoimmune encephalomyelitis; MPTP, mitochondrial permeability transition pore; NMDA, *N*-methyl-d-aspartate; PBI, primary blast injury; ROCK, Rho-associated protein kinase; STZ, streptozotocin; TUNEL, terminal deoxynucleotidyl transferase dUTP nick end labelling.

## References

[bib1] Berry M , Ahmed Z , Lorber B , Douglas M , Logan A . Regeneration of axons in the visual system. Restor Neurol Neurosci 2008; 26: 147–174. 18820408

[bib2] Nicholson DW . Caspase structure, proteolytic substrates, and function during apoptotic cell death. Cell Death Differ 1999; 6: 1028–1042. 10578171 10.1038/sj.cdd.4400598

[bib3] Nicholson DW , Thornberry NA . Caspases: killer proteases. Trends Biochem Sci 1997; 22: 299–306. 9270303 10.1016/s0968-0004(97)01085-2

[bib4] Jimenez Fernandez D , Lamkanfi M . Inflammatory caspases: key regulators of inflammation and cell death. Biol Chem 2015; 396: 193–203. 25389992 10.1515/hsz-2014-0253

[bib5] Fan T-J , Han L-H , Cong R-S , Liang J . Caspase family proteases and apoptosis. Acta Biochim Biophys Sin (Shanghai) 2005; 37: 719–727. 16270150 10.1111/j.1745-7270.2005.00108.x

[bib6] Parrish AB , Freel CD , Kornbluth S . Cellular mechanisms controlling caspase activation and function. Cold Spring Harb Perspect Biol 2013; 5. 10.1101/cshperspect.a008672 PMC3660825 23732469

[bib7] Cullen SP , Martin SJ . Caspase activation pathways: some recent progress. Cell Death Differ 2009; 16: 935–938. 19528949 10.1038/cdd.2009.59

[bib8] Kumar S . Caspase function in programmed cell death. Cell Death Differ 2007; 14: 32–43. 17082813 10.1038/sj.cdd.4402060

[bib9] Kischkel FC , Hellbardt S , Behrmann I , Germer M , Pawlita M , Krammer PH et al. Cytotoxicity-dependent Apo-1 (Fas/Cd95)-associated proteins form a death-inducing signaling complex (Disc) with the receptor. EMBO J 1995; 14: 5579–5588. 8521815 10.1002/j.1460-2075.1995.tb00245.x PMC394672

[bib10] Chinnaiyan AM , O'Rourke K , Tewari M , Dixit VM . FADD, a novel death domain-containing protein, interacts with the death domain of Fas and initiates apoptosis. Cell 1995; 81: 505–512. 7538907 10.1016/0092-8674(95)90071-3

[bib11] Wajant H . The Fas signaling pathway: more than a paradigm. Science 2002; 296: 1635–1636. 12040174 10.1126/science.1071553

[bib12] Muzio M . Signalling by proteolysis: death receptors induce apoptosis. Int J Clin Lab Res 1998; 28: 141–147. 9801924 10.1007/s005990050035

[bib13] Kuwana T , Smith JJ , Muzio M , Dixit V , Newmeyer DD , Kornbluth S . Apoptosis induction by caspase-8 is amplified through the mitochondrial release of cytochrome c. J Biol Chem 1998; 273: 16589–16594. 9632731 10.1074/jbc.273.26.16589

[bib14] Kroemer G , Galluzzi L , Brenner C . Mitochondrial membrane permeabilization in cell death. Physiol Rev 2007; 87: 99–163. 17237344 10.1152/physrev.00013.2006

[bib15] Nickells RW . Variations in the rheostat model of apoptosis: what studies of retinal ganglion cell death tell us about the functions of the Bcl2 family proteins. Exp Eye Res 2010; 91: 2–8. 20230818 10.1016/j.exer.2010.03.004 PMC2885977

[bib16] Li P , Nijhawan D , Budihardjo I , Srinivasula SM , Ahmad M , Alnemri ES et al. Cytochrome c and dATP-dependent formation of Apaf-1/caspase-9 complex initiates an apoptotic protease cascade. Cell 1997; 91: 479–489. 9390557 10.1016/s0092-8674(00)80434-1

[bib17] Adams JM , Cory S . Apoptosomes: engines for caspase activation. Curr Opin Cell Biol 2002; 14: 715–720. 12473344 10.1016/s0955-0674(02)00381-2

[bib18] Vanden Berghe T , Linkermann A , Jouan-Lanhouet S , Walczak H , Vandenabeele P . Regulated necrosis: the expanding network of non-apoptotic cell death pathways. Nat Rev Mol Cell Biol 2014; 15: 135–147. 24452471 10.1038/nrm3737

[bib19] Micheau O , Tschopp J . Induction of TNF receptor I-mediated apoptosis via two sequential signaling complexes. Cell 2003; 114: 181–190. 12887920 10.1016/s0092-8674(03)00521-x

[bib20] Feltham R , Vince JE , Lawlor KE . Caspase-8: not so silently deadly. Clin Transl Immunology 2017; 6: e124. 28197335 10.1038/cti.2016.83 PMC5292560

[bib21] Dondelinger Y , Declercq W , Montessuit S , Roelandt R , Goncalves A , Bruggeman I et al. MLKL compromises plasma membrane integrity by binding to phosphatidylinositol phosphates. Cell Rep 2014; 7: 971–981. 24813885 10.1016/j.celrep.2014.04.026

[bib22] Scott FL , Denault JB , Riedl SJ , Shin H , Renatus M , Salvesen GS . XIAP inhibits caspase-3 and -7 using two binding sites: evolutionarily conserved mechanism of IAPs. EMBO J 2005; 24: 645–655. 15650747 10.1038/sj.emboj.7600544 PMC548652

[bib23] Tenev T , Bianchi K , Darding M , Broemer M , Langlais C , Wallberg F et al. The Ripoptosome, a signaling platform that assembles in response to genotoxic stress and loss of IAPs. Mol Cell 2011; 43: 432–448. 21737329 10.1016/j.molcel.2011.06.006

[bib24] Feoktistova M , Geserick P , Panayotova-Dimitrova D , Leverkus M . Pick your poison: the Ripoptosome, a cell death platform regulating apoptosis and necroptosis. Cell Cycle 2012; 11: 460–467. 22274400 10.4161/cc.11.3.19060

[bib25] Henry CM , Martin SJ . Caspase-8 acts in a non-enzymatic role as a scaffold for assembly of a pro-inflammatory "FADDosome" complex upon TRAIL stimulation. Mol Cell 2017; 65: 715–29 e5. 28212752 10.1016/j.molcel.2017.01.022

[bib26] Guo Y , Srinivasula SM , Druilhe A , Fernandes-Alnemri T , Alnemri ES . Caspase-2 induces apoptosis by releasing proapoptotic proteins from mitochondria. J Biol Chem 2002; 277: 13430–13437. 11832478 10.1074/jbc.M108029200

[bib27] Lassus P , Opitz-Araya X , Lazebnik Y . Requirement for caspase-2 in stress-induced apoptosis before mitochondrial permeabilization. Science 2002; 297: 1352–1354. 12193789 10.1126/science.1074721

[bib28] Ho LH , Read SH , Dorstyn L , Lambrusco L , Kumar S . Caspase-2 is required for cell death induced by cytoskeletal disruption. Oncogene 2008; 27: 3393–3404. 18193089 10.1038/sj.onc.1211005

[bib29] Tu S , McStay GP , Boucher LM , Mak T , Beere HM , Green DR . In situ trapping of activated initiator caspases reveals a role for caspase-2 in heat shock-induced apoptosis. Nat Cell Biol 2006; 8: 72–U24. 16362053 10.1038/ncb1340

[bib30] Tinel A , Tschopp J . The PIDDosome, a protein complex implicated in activation of caspase-2 in response to genotoxic stress. Science 2004; 304: 843–846. 15073321 10.1126/science.1095432

[bib31] Sidi S , Sanda T , Kennedy RD , Hagen AT , Jette CA , Hoffmans R et al. Chk1 suppresses a caspase-2 apoptotic response to DNA damage that bypasses p53, Bcl-2, and caspase-3. Cell 2008; 133: 864–877. 18510930 10.1016/j.cell.2008.03.037 PMC2719897

[bib32] Upton JP , Austgen K , Nishino M , Coakley KM , Hagen A , Han D et al. Caspase-2 cleavage of BID is a critical apoptotic signal downstream of endoplasmic reticulum stress. Mol Cell Biol 2008; 28: 3943–3951. 18426910 10.1128/MCB.00013-08 PMC2423129

[bib33] Duan H , Dixit VM . RAIDD is a new 'death' adaptor molecule. Nature 1997; 385: 86–89. 8985253 10.1038/385086a0

[bib34] Lin Y , Ma W , Benchimol S . Pidd, a new death-domain-containing protein, is induced by p53 and promotes apoptosis. Nat Genet 2000; 26: 122–127. 10973264 10.1038/79102

[bib35] Bouchier-Hayes L , Green DR . Caspase-2: the orphan caspase. Cell Death Differ 2012; 19: 51–57. 22075987 10.1038/cdd.2011.157 PMC3252831

[bib36] Lopez-Cruzan M , Sharma R , Tiwari M , Karbach S , Holstein D , Martin CR et al. Caspase-2 resides in the mitochondria and mediates apoptosis directly from the mitochondrial compartment. Cell Death Discov 2016; 2: 16005. 27019748 10.1038/cddiscovery.2016.5 PMC4806400

[bib37] Vanaja SK , Rathinam VA , Fitzgerald KA . Mechanisms of inflammasome activation: recent advances and novel insights. Trends Cell Biol 2015; 25: 308–315. 25639489 10.1016/j.tcb.2014.12.009 PMC4409512

[bib38] Broz P , Dixit VM . Inflammasomes: mechanism of assembly, regulation and signalling. Nat Rev Immunol 2016; 16: 407–420. 27291964 10.1038/nri.2016.58

[bib39] Franchi L , Eigenbrod T , Munoz-Planillo R , Nunez G . The inflammasome: a caspase-1-activation platform that regulates immune responses and disease pathogenesis. Nat Immunol 2009; 10: 241–247. 19221555 10.1038/ni.1703 PMC2820724

[bib40] Kayagaki N , Stowe IB , Lee BL , O'Rourke K , Anderson K , Warming S et al. Caspase-11 cleaves gasdermin D for non-canonical inflammasome signalling. Nature 2015; 526: 666–671. 26375259 10.1038/nature15541

[bib41] Shi J , Zhao Y , Wang K , Shi X , Wang Y , Huang H et al. Cleavage of GSDMD by inflammatory caspases determines pyroptotic cell death. Nature 2015; 526: 660–665. 26375003 10.1038/nature15514

[bib42] He WT , Wan H , Hu L , Chen P , Wang X , Huang Z et al. Gasdermin D is an executor of pyroptosis and required for interleukin-1beta secretion. Cell Res 2015; 25: 1285–1298. 26611636 10.1038/cr.2015.139 PMC4670995

[bib43] Menu P , Vince JE . The NLRP3 inflammasome in health and disease: the good, the bad and the ugly. Clin Exp Immunol 2011; 166: 1–15. 21762124 10.1111/j.1365-2249.2011.04440.x PMC3193914

[bib44] Munoz-Planillo R , Kuffa P , Martinez-Colon G , Smith BL , Rajendiran TM , Nunez G . K(+) efflux is the common trigger of NLRP3 inflammasome activation by bacterial toxins and particulate matter. Immunity 2013; 38: 1142–1153. 23809161 10.1016/j.immuni.2013.05.016 PMC3730833

[bib45] Gaidt MM , Ebert TS , Chauhan D , Schmidt T , Schmid-Burgk JL , Rapino F et al. Human monocytes engage an alternative inflammasome pathway. Immunity 2016; 44: 833–846. 27037191 10.1016/j.immuni.2016.01.012

[bib46] Wolf AJ , Reyes CN , Liang W , Becker C , Shimada K , Wheeler ML et al. Hexokinase is an innate immune receptor for the detection of bacterial peptidoglycan. Cell 2016; 166: 624–636. 27374331 10.1016/j.cell.2016.05.076 PMC5534359

[bib47] Yang D , He Y , Munoz-Planillo R , Liu Q , Nunez G . Caspase-11 requires the pannexin-1 channel and the purinergic P2X7 pore to mediate pyroptosis and endotoxic shock. Immunity 2015; 43: 923–932. 26572062 10.1016/j.immuni.2015.10.009 PMC4795157

[bib48] Lawlor KE , Khan N , Mildenhall A , Gerlic M , Croker BA , D'Cruz AA et al. RIPK3 promotes cell death and NLRP3 inflammasome activation in the absence of MLKL. Nat Commun 2015; 6: 6282. 25693118 10.1038/ncomms7282 PMC4346630

[bib49] Allam R , Lawlor KE , Yu ECW , Mildenhall AL , Moujalled DM , Lewis RS et al. Mitochondrial apoptosis is dispensable for NLRP3 inflammasome activation but non-apoptotic caspase-8 is required for inflammasome priming. EMBO Rep 2014; 15: 982–990. 24990442 10.15252/embr.201438463 PMC4198042

[bib50] Gutierrez KD , Davis MA , Daniels BP , Olsen TM , Ralli-Jain P , Tait SW et al. MLKL activation triggers NLRP3-mediated processing and release of IL-1beta independently of gasdermin-D. J Immunol 2017; 198: 2156–2164. 28130493 10.4049/jimmunol.1601757 PMC5321867

[bib51] Rozman-Pungercar J , Kopitar-Jerala N , Bogyo M , Turk D , Vasiljeva O , Stefe I et al. Inhibition of papain-like cysteine proteases and legumain by caspase-specific inhibitors: when reaction mechanism is more important than specificity. Cell Death Differ 2003; 10: 881–888. 12867995 10.1038/sj.cdd.4401247

[bib52] Caserta TM , Smith AN , Gultice AD , Reedy MA , Brown TL . Q-VD-OPh, a broad spectrum caspase inhibitor with potent antiapoptotic properties. Apoptosis 2003; 8: 345–352. 12815277 10.1023/a:1024116916932

[bib53] Callus BA , Vaux DL . Caspase inhibitors: viral, cellular and chemical. Cell Death Differ 2007; 14: 73–78. 16946729 10.1038/sj.cdd.4402034

[bib54] Ekert PG , Silke J , Vaux DL . Caspase inhibitors. Cell Death Differ 1999; 6: 1081–1086. 10578177 10.1038/sj.cdd.4400594

[bib55] McStay GP , Salvesen GS , Green DR . Overlapping cleavage motif selectivity of caspases: implications for analysis of apoptotic pathways. Cell Death Differ 2008; 15: 322–331. 17975551 10.1038/sj.cdd.4402260

[bib56] Chauvier D , Ankri S , Charriaut-Marlangue C , Casimir R , Jacotot E . Broad-spectrum caspase inhibitors: from myth to reality? Cell Death Differ 2007; 14: 387–391. 17008913 10.1038/sj.cdd.4402044

[bib57] Schotte P , Declercq W , Van Huffel S , Vandenabeele P , Beyaert R . Non-specific effects of methyl ketone peptide inhibitors of caspases. FEBS Lett 1999; 442: 117–121. 9923616 10.1016/s0014-5793(98)01640-8

[bib58] Berger AB , Sexton KB , Bogyo M . Commonly used caspase inhibitors designed based on substrate specificity profiles lack selectivity. Cell Res 2006; 16: 961–963. 17117159 10.1038/sj.cr.7310112

[bib59] Pereira NA , Song Z . Some commonly used caspase substrates and inhibitors lack the specificity required to monitor individual caspase activity. Biochem Biophys Res Commun 2008; 377: 873–877. 18976637 10.1016/j.bbrc.2008.10.101

[bib60] Jackson AL , Linsley PS . Recognizing and avoiding siRNA off-target effects for target identification and therapeutic application. Nat Rev Drug Discov 2010; 9: 57–67. 20043028 10.1038/nrd3010

[bib61] Monnier PP , D'Onofrio PM , Magharious M , Hollander AC , Tassew N , Szydlowska K et al. Involvement of caspase-6 and caspase-8 in neuronal apoptosis and the regenerative failure of injured retinal ganglion cells. J Neurosci 2011; 31: 10494–10505. 21775595 10.1523/JNEUROSCI.0148-11.2011 PMC6622648

[bib62] Patil K , Sharma SC . Broad spectrum caspase inhibitor rescues retinal ganglion cells after ischemia. Neuroreport 2004; 15: 981–984. 15076719 10.1097/00001756-200404290-00010

[bib63] Bahr M . Live or let die - retinal ganglion cell death and survival during development and in the lesioned adult CNS. Trends Neurosci 2000; 23: 483–490. 11006465 10.1016/s0166-2236(00)01637-4

[bib64] Cellerino A , Bahr M , Isenmann S . Apoptosis in the developing visual system. Cell Tissue Res 2000; 301: 53–69. 10928281 10.1007/s004410000178

[bib65] Perry VH , Henderson Z , Linden R . Postnatal changes in retinal ganglion cell and optic axon populations in the pigmented rat. J Comp Neurol 1983; 219: 356–368. 6619343 10.1002/cne.902190309

[bib66] Chavarria T , Baleriola J , Mayordomo R , de Pablo F , de la Rosa EJ . Early neural cell death is an extensive, dynamic process in the embryonic chick and mouse retina. ScientificWorldJournal 2013; 2013: 627240. 23710143 10.1155/2013/627240 PMC3654239

[bib67] Mayordomo R , Valenciano AI , de la Rosa EJ , Hallbook F . Generation of retinal ganglion cells is modulated by caspase-dependent programmed cell death. Eur J Neurosci 2003; 18: 1744–1750. 14622209 10.1046/j.1460-9568.2003.02891.x

[bib68] Ding Q , Chen H , Xie X , Libby RT , Tian N , Gan L . BARHL2 differentially regulates the development of retinal amacrine and ganglion neurons. J Neurosci 2009; 29: 3992–4003. 19339595 10.1523/JNEUROSCI.5237-08.2009 PMC2756297

[bib69] Kuida K , Zheng TS , Na S , Kuan C , Yang D , Karasuyama H et al. Decreased apoptosis in the brain and premature lethality in CPP32-deficient mice. Nature 1996; 384: 368–372. 8934524 10.1038/384368a0

[bib70] Hakem R , Hakem A , Duncan GS , Henderson JT , Woo M , Soengas MS et al. Differential requirement for caspase 9 in apoptotic pathways *in vivo*. Cell 1998; 94: 339–352. 9708736 10.1016/s0092-8674(00)81477-4

[bib71] Kuida K , Haydar TF , Kuan CY , Gu Y , Taya C , Karasuyama H et al. Reduced apoptosis and cytochrome c-mediated caspase activation in mice lacking caspase 9. Cell 1998; 94: 325–337. 9708735 10.1016/s0092-8674(00)81476-2

[bib72] Kumar S , Tomooka Y , Noda M . Identification of a set of genes with developmentally down-regulated expression in the mouse brain. Biochem Biophys Res Commun 1992; 185: 1155–1161. 1378265 10.1016/0006-291x(92)91747-e

[bib73] Kojima M , Asahi M , Kikuchi H , Hashimoto N , Noda M , Hoshimaru M . Expression of Nedd2/ICH-1 (caspase-2) in the developing rat retina. Neurosci Res 1998; 31: 211–217. 9809666 10.1016/s0168-0102(98)00039-x

[bib74] Kisiswa L , Albon J , Morgan JE , Wride MA . Cellular inhibitor of apoptosis (cIAP1) is down-regulated during retinal ganglion cell (RGC) maturation. Exp Eye Res 2010; 91: 739–747. 20831867 10.1016/j.exer.2010.08.024

[bib75] Compston A , Coles A . Multiple sclerosis. Lancet 2008; 372: 1502–1517. 18970977 10.1016/S0140-6736(08)61620-7

[bib76] Talman LS , Bisker ER , Sackel DJ , Long DA Jr. , Galetta KM , Ratchford JN et al. Longitudinal study of vision and retinal nerve fiber layer thickness in multiple sclerosis. Ann Neurol 2010; 67: 749–760. 20517936 10.1002/ana.22005 PMC2901775

[bib77] Meyer R , Weissert R , Diem R , Storch MK , de Graaf KL , Kramer B et al. Acute neuronal apoptosis in a rat model of multiple sclerosis. J Neurosci 2001; 21: 6214–6220. 11487644 10.1523/JNEUROSCI.21-16-06214.2001 PMC6763179

[bib78] Horstmann L , Schmid H , Heinen AP , Kurschus FC , Dick HB , Joachim SC . Inflammatory demyelination induces glia alterations and ganglion cell loss in the retina of an experimental autoimmune encephalomyelitis model. J Neuroinflammation 2013; 10: 120. 24090415 10.1186/1742-2094-10-120 PMC3851328

[bib79] Talla V , Koilkonda R , Porciatti V , Chiodo V , Boye SL , Hauswirth WW et al. Complex I subunit gene therapy with NDUFA6 ameliorates neurodegeneration in EAE. Invest Ophthalmol Vis Sci 2015; 56: 1129–1140. 25613946 10.1167/iovs.14-15950 PMC4329968

[bib80] Sattler MB , Merkler D , Maier K , Stadelmann C , Ehrenreich H , Bahr M et al. Neuroprotective effects and intracellular signaling pathways of erythropoietin in a rat model of multiple sclerosis. Cell Death Differ 2004; 11 (Suppl 2): S181–S192. 15459752 10.1038/sj.cdd.4401504

[bib81] Lidster K , Jackson SJ , Ahmed Z , Munro P , Coffey P , Giovannoni G et al. Neuroprotection in a novel mouse model of multiple sclerosis. PLoS ONE 2013; 8: e79188. 24223903 10.1371/journal.pone.0079188 PMC3817036

[bib82] Wu N , Yin ZQ , Wang Y . Traumatic optic neuropathy therapy: an update of clinical and experimental studies. J Int Med Res 2008; 36: 883–889. 18831880 10.1177/147323000803600503

[bib83] Wormald R , Dickersin K , Cochrane E , Vision G . Evidence-based ophthalmology. Ophthalmology 2013; 120: 2361–3 e1. 24246821 10.1016/j.ophtha.2013.08.032

[bib84] Levin LA , Beck RW , Joseph MP , Seiff S , Kraker R . The treatment of traumatic optic neuropathy: the International Optic Nerve Trauma Study. Ophthalmology 1999; 106: 1268–1277. 10406604 10.1016/s0161-6420(99)00707-1

[bib85] Sarkies N . Traumatic optic neuropathy. Eye (Lond) 2004; 18: 1122–1125. 15534597 10.1038/sj.eye.6701571

[bib86] Berry M , Carlile J , Hunter A . Peripheral nerve explants grafted into the vitreous body of the eye promote the regeneration of retinal ganglion cell axons severed in the optic nerve. J Neurocytol 1996; 25: 147–170. 8699196 10.1007/BF02284793

[bib87] Berry M , Carlile J , Hunter A , Tsang W , Rosenstiel P , Rosustrel P et al. Optic nerve regeneration after intravitreal peripheral nerve implants: trajectories of axons regrowing through the optic chiasm into the optic tracts. J Neurocytol 1999; 28: 721–741. 10859575 10.1023/a:1007086004022

[bib88] Villegas-Perez MP , Vidal-Sanz M , Rasminsky M , Bray GM , Aguayo AJ . Rapid and protracted phases of retinal ganglion cell loss follow axotomy in the optic nerve of adult rats. J Neurobiol 1993; 24: 23–36. 8419522 10.1002/neu.480240103

[bib89] Berkelaar M , Clarke DB , Wang YC , Bray GM , Aguayo AJ . Axotomy results in delayed death and apoptosis of retinal ganglion cells in adult rats. J Neurosci 1994; 14: 4368–4374. 8027784 10.1523/JNEUROSCI.14-07-04368.1994 PMC6577016

[bib90] Cheung ZH , Chan YM , Siu FK , Yip HK , Wu W , Leung MC et al. Regulation of caspase activation in axotomized retinal ganglion cells. Mol Cell Neurosci 2004; 25: 383–393. 15033167 10.1016/j.mcn.2003.11.001

[bib91] Ahmed Z , Kalinski H , Berry M , Almasieh M , Ashush H , Slager N et al. Ocular neuroprotection by siRNA targeting caspase-2. Cell Death Dis 2011; 2: e173. 21677688 10.1038/cddis.2011.54 PMC3168996

[bib92] Agudo M , Perez-Marin MC , Lonngren U , Sobrado P , Conesa A , Canovas I et al. Time course profiling of the retinal transcriptome after optic nerve transection and optic nerve crush. Mol Vis 2008; 14: 1050–1063. 18552980 PMC2426719

[bib93] Garcia-Valenzuela E , Gorczyca W , Darzynkiewicz Z , Sharma SC . Apoptosis in adult retinal ganglion cells after axotomy. J Neurobiol 1994; 25: 431–438. 8077968 10.1002/neu.480250408

[bib94] Rabacchi SA , Bonfanti L , Liu XH , Maffei L . Apoptotic cell-death induced by optic-nerve lesion in the neonatal rat. J Neurosci 1994; 14: 5292–5301. 8083737 10.1523/JNEUROSCI.14-09-05292.1994 PMC6577062

[bib95] Vigneswara V , Berry M , Logan A , Ahmed Z . Pharmacological inhibition of caspase-2 protects axotomised retinal ganglion cells from apoptosis in adult rats. PLoS ONE 2012; 7: e53473. 23285297 10.1371/journal.pone.0053473 PMC3532067

[bib96] Vigneswara V , Akpan N , Berry M , Logan A , Troy CM , Ahmed Z . Combined suppression of CASP2 and CASP6 protects retinal ganglion cells from apoptosis and promotes axon regeneration through CNTF-mediated JAK/STAT signalling. Brain 2014; 137 (Pt 6): 1656–1675. 24727569 10.1093/brain/awu037 PMC4032097

[bib97] Weishaupt JH , Diem R , Kermer P , Krajewski S , Reed JC , Bahr M . Contribution of caspase-8 to apoptosis of axotomized rat retinal ganglion cells *in vivo*. Neurobiol Dis 2003; 13: 124–135. 12828936 10.1016/s0969-9961(03)00032-9

[bib98] Grosskreutz CL , Hanninen VA , Pantcheva MB , Huang W , Poulin NR , Dobberfuhl AP . FK506 blocks activation of the intrinsic caspase cascade after optic nerve crush. Exp Eye Res 2005; 80: 681–686. 15862175 10.1016/j.exer.2004.11.017

[bib99] Kermer P , Ankerhold R , Klocker N , Krajewski S , Reed JC , Bahr M . Caspase-9: involvement in secondary death of axotomized rat retinal ganglion cells *in vivo*. Brain Res Mol Brain Res 2000; 85: 144–150. 11146116 10.1016/s0169-328x(00)00256-4

[bib100] Kermer P , Klocker N , Bahr M . Long-term effect of inhibition of ced 3-like caspases on the survival of axotomized retinal ganglion cells *in vivo*. Exp Neurol 1999; 158: 202–205. 10448432 10.1006/exnr.1999.7094

[bib101] Sanchez-Migallon MC , Valiente-Soriano FJ , Nadal-Nicolas FM , Vidal-Sanz M , Agudo-Barriuso M . Apoptotic retinal ganglion cell death after optic nerve transection or crush in mice: delayed RGC loss with BDNF or a caspase 3 inhibitor. Invest Ophthalmol Vis Sci 2016; 57: 81–93. 26780312 10.1167/iovs.15-17841

[bib102] Chaudhary P , Ahmed F , Quebada P , Sharma SC . Caspase inhibitors block the retinal ganglion cell death following optic nerve transection. Brain Res Mol Brain Res 1999; 67: 36–45. 10101230 10.1016/s0169-328x(99)00032-7

[bib103] Kermer P , Klocker N , Labes M , Thomsen S , Srinivasan A , Bahr M . Activation of caspase-3 in axotomized rat retinal ganglion cells *in vivo*. FEBS Lett 1999; 453: 361–364. 10405176 10.1016/s0014-5793(99)00747-4

[bib104] He MH , Cheung ZH , Yu EH , Tay DK , So KF . Cytochrome c release and caspase-3 activation in retinal ganglion cells following different distance of axotomy of the optic nerve in adult hamsters. Neurochem Res 2004; 29: 2153–2161. 15662850 10.1007/s11064-004-6889-6

[bib105] Levkovitch-Verbin H , Dardik R , Vander S , Melamed S . Mechanism of retinal ganglion cells death in secondary degeneration of the optic nerve. Exp Eye Res 2010; 91: 127–134. 19951705 10.1016/j.exer.2009.11.014

[bib106] Choudhury S , Liu Y , Clark AF , Pang IH . Caspase-7: a critical mediator of optic nerve injury-induced retinal ganglion cell death. Mol Neurodegener 2015; 10: 40. 26306916 10.1186/s13024-015-0039-2 PMC4550044

[bib107] Agudo M , Perez-Marin MC , Sobrado-Calvo P , Lonngren U , Salinas-Navarro M , Canovas I et al. Immediate upregulation of proteins belonging to different branches of the apoptotic cascade in the retina after optic nerve transection and optic nerve crush. Invest Ophthalmol Vis Sci 2009; 50: 424–431. 18775855 10.1167/iovs.08-2404

[bib108] Puyang Z , Feng L , Chen H , Liang P , Troy JB , Liu X . Retinal ganglion cell loss is delayed following optic nerve crush in NLRP3 knockout mice. Sci Rep 2016; 6: 20998. 26893104 10.1038/srep20998 PMC4759563

[bib109] Kermer P , Klöcker N , Bähr M . Long-term effect of inhibition of CED 3-like caspases on the survival of axotomized retinal ganglion cells *in vivo*. Exp Neurol 1999; 158: 202–205. 10448432 10.1006/exnr.1999.7094

[bib110] Liu Y , Yan H , Chen S , Sabel BA . Caspase-3 inhibitor Z-DEVD-FMK enhances retinal ganglion cell survival and vision restoration after rabbit traumatic optic nerve injury. Restor Neurol Neurosci 2015; 33: 205–220. 25588462 10.3233/RNN-159001

[bib111] Tura A , Schuettauf F , Monnier PP , Bartz-Schmidt KU , Henke-Fahle S . Efficacy of Rho-kinase inhibition in promoting cell survival and reducing reactive gliosis in the rodent retina. Invest Ophthalmol Vis Sci 2009; 50: 452–461. 18757509 10.1167/iovs.08-1973

[bib112] Koch JC , Tonges L , Barski E , Michel U , Bahr M , Lingor P . ROCK2 is a major regulator of axonal degeneration, neuronal death and axonal regeneration in the CNS. Cell Death Dis 2014; 5: e1225. 24832597 10.1038/cddis.2014.191 PMC4047920

[bib113] Zhang ZZ , Gong YY , Shi YH , Zhang W , Qin XH , Wu XW . Valproate promotes survival of retinal ganglion cells in a rat model of optic nerve crush. Neuroscience 2012; 224: 282–293. 22867974 10.1016/j.neuroscience.2012.07.056

[bib114] Zhang ZZ , Qin XH , Tong NT , Zhao XF , Gong YY , Shi YH et al. Valproic acid-mediated neuroprotection in retinal ischemia injury via histone deacetylase inhibition and transcriptional activation. Exp Eye Res 2012; 94: 98–108. 22143029 10.1016/j.exer.2011.11.013

[bib115] Liu Y , Yan H , Chen S , Sabel BA . Caspase-3 inhibitor Z-DEVD-FMK enhances retinal ganglion cell survival and vision restoration after rabbit traumatic optic nerve injury. Restor Neurol Neurosci 2015; 33: 205–220. 25588462 10.3233/RNN-159001

[bib116] Vigneswara V , Ahmed Z . Long-term neuroprotection of retinal ganglion cells by inhibiting caspase-2. Cell Death Discov 2016; 2: 16044. 27551534 10.1038/cddiscovery.2016.44 PMC4979513

[bib117] Vigneswara V , Berry M , Logan A , Ahmed Z . Caspase-2 is upregulated after sciatic nerve transection and its inhibition protects dorsal root ganglion neurons from apoptosis after serum withdrawal. PLoS ONE 2013; 8: e57861. 23451279 10.1371/journal.pone.0057861 PMC3581492

[bib118] Nadal-Nicolas FM , Galindo-Romero C , Valiente-Soriano FJ , Barbera-Cremades M , deTorre-Minguela C , Salinas-Navarro M et al. Involvement of P2X7 receptor in neuronal degeneration triggered by traumatic injury. Sci Rep 2016; 6: 38499. 27929040 10.1038/srep38499 PMC5144087

[bib119] Blanch RJ , Scott RAH . Primary blast injury of the eye. J R Army Medical Corps 2008; 154: 76. 19093301

[bib120] Scott R . The injured eye. Philos T R Soc B 2011; 366: 251–260. 10.1098/rstb.2010.0234 PMC3013431 21149360

[bib121] Cockerham GC , Rice TA , Hewes EH , Cockerham KP , Lemke S , Wang G et al. Closed-eye ocular injuries in the Iraq and Afghanistan wars. N Engl J Med 2011; 364: 2172–2173. 21631351 10.1056/NEJMc1010683

[bib122] Warden D . Military TBI during the Iraq and Afghanistan wars. J Head Trauma Rehab 2006; 21: 398–402. 10.1097/00001199-200609000-00004 16983225

[bib123] Wang HCH , Choi JH , Greene WA , Plamper ML , Cortez HE , Chavko M et al. Pathophysiology of blast-induced ocular trauma with apoptosis in the retina and optic nerve. Mil Med 2014; 179: 34–40. 25102547 10.7205/MILMED-D-13-00504

[bib124] Choi JH , Greene WA , Johnson AJ , Chavko M , Cleland JM , McCarron RM et al. Pathophysiology of blast-induced ocular trauma in rats after repeated exposure to low-level blast overpressure. Clin Exp Ophthalmol 2015; 43: 239–246. 25112787 10.1111/ceo.12407

[bib125] Jiang YD , Liu L , Pagadala J , Miller DD , Steinle JJ . Compound 49b protects against blast-induced retinal injury. J Neuroinflammation 2013; 10: 96. 23899290 10.1186/1742-2094-10-96 PMC3751549

[bib126] Weichel ED , Colyer MH , Ludlow SE , Bower KS , Eiseman AS . Combat ocular trauma visual outcomes during Operations Iraqi and Enduring Freedom. Ophthalmology 2008; 115: 2235–2245. 19041478 10.1016/j.ophtha.2008.08.033

[bib127] Zou YY , Kan EM , Lu J , Ng KC , Tan MH , Yao LL et al. Primary blast injury-induced lesions in the retina of adult rats. J Neuroinflammation 2013; 10: 79. 23819902 10.1186/1742-2094-10-79 PMC3707737

[bib128] Mo JS , Anderson MG , Gregory M , Smith RS , Savinova OV , Serreze DV et al. By altering ocular immune privilege, bone marrow-derived cells pathogenically contribute to DBA/2J pigmentary glaucoma. J Exp Med 2003; 197: 1335–1344. 12756269 10.1084/jem.20022041 PMC2193785

[bib129] Bricker-Anthony C , Rex TS . Neurodegeneration and vision loss after mild blunt trauma in the C57Bl/6 and DBA/2J mouse. PLoS ONE 2015; 10: e0131921. 26148200 10.1371/journal.pone.0131921 PMC4493046

[bib130] Bricker-Anthony C , Hines-Beard J , D'Surney L , Rex TS . Exacerbation of blast-induced ocular trauma by an immune response. J Neuroinflammation 2014; 11: 192. 25472427 10.1186/s12974-014-0192-5 PMC4264554

[bib131] Mohan K , Kecova H , Hernandez-Merino E , Kardon RH , Harper MM . Retinal ganglion cell damage in an experimental rodent model of blast-mediated traumatic brain injury. Invest Ophthalmol Vis Sci 2013; 54: 3440–3450. 23620426 10.1167/iovs.12-11522 PMC4597486

[bib132] Dutca LM , Stasheff SF , Hedberg-Buenz A , Rudd DS , Batra N , Blodi FR et al. Early detection of subclinical visual damage after blast-mediated TBI enables prevention of chronic visual deficit by treatment with P7C3-S243. Invest Ophthalmol Vis Sci 2014; 55: 8330–8341. 25468886 10.1167/iovs.14-15468 PMC5102342

[bib133] Warner N , Eggenberger E . Traumatic optic neuropathy: a review of the current literature. Curr Opin Ophthalmol 2010; 21: 459–462. 20829687 10.1097/ICU.0b013e32833f00c9

[bib134] Lam TT , Abler AS , Kwong JM , Tso MO . N-methyl-D-aspartate (NMDA)--induced apoptosis in rat retina. Invest Ophthalmol Vis Sci 1999; 40: 2391–2397. 10476807

[bib135] Schuettauf F , Stein T , Choragiewicz TJ , Rejdak R , Bolz S , Zurakowski D et al. Caspase inhibitors protect against NMDA-mediated retinal ganglion cell death. Clin Exp Ophthalmol 2011; 39: 545–554. 21176044 10.1111/j.1442-9071.2010.02486.x

[bib136] Seki M , Soussou W , Manabe S , Lipton SA . Protection of retinal ganglion cells by caspase substrate-binding peptide IQACRG from N-methyl-D-aspartate receptor-mediated excitotoxicity. Invest Ophthalmol Vis Sci 2010; 51: 1198–1207. 19815732 10.1167/iovs.09-4102 PMC2868456

[bib137] Sui GY , Liu GC , Liu GY , Gao YY , Deng Y , Wang WY et al. Is sunlight exposure a risk factor for age-related macular degeneration? A systematic review and meta-analysis. Br J Ophthalmol 2013; 97: 389–394. 23143904 10.1136/bjophthalmol-2012-302281

[bib138] Fletcher AE , Bentham GC , Agnew M , Young IS , Augood C , Chakravarthy U et al. Sunlight exposure, antioxidants, and age-related macular degeneration. Arch Ophthalmol 2008; 126: 1396–1403. 18852418 10.1001/archopht.126.10.1396

[bib139] Marc RE , Jones BW , Watt CB , Vazquez-Chona F , Vaughan DK , Organisciak DT . Extreme retinal remodeling triggered by light damage: implications for age related macular degeneration. Mol Vis 2008; 14: 782–806. 18483561 PMC2375357

[bib140] Shu QM , Xu Y , Zhuang H , Fan JW , Sun ZC , Zhang M et al. Ras homolog enriched in the brain is linked to retinal ganglion cell apoptosis after light injury in rats. J Mol Neurosci 2014; 54: 243–251. 24664437 10.1007/s12031-014-0281-z

[bib141] Yang X , Chen H , Zhu M , Zhu R , Qin B , Fang H et al. Up-regulation of PKM2 relates to retinal ganglion cell apoptosis after light-induced retinal damage in adult rats. Cell Mol Neurobiol 2015; 35: 1175–1186. 25990228 10.1007/s10571-015-0211-9 PMC11486339

[bib142] Sang A , Yang X , Chen H , Qin B , Zhu M , Dai M et al. Upregulation of SYF2 relates to retinal ganglion cell apoptosis and retinal glia cell proliferation after light-induced retinal damage. J Mol Neurosci 2015; 56: 480–490. 25944718 10.1007/s12031-015-0534-5

[bib143] Xu Y , Yu SS , Shu QM , Yang L , Yang C , Wang JW et al. Upregulation of CREM-1 relates to retinal ganglion cells apoptosis after light-induced damage *in vivo*. J Mol Neurosci 2014; 52: 331–338. 24166353 10.1007/s12031-013-0153-y

[bib144] Sang A , Xu Y , Jin N , Zhou T , Wang J , Zhu J et al. Involvement of transcription initiation factor IIB in the light-induced death of rat retinal ganglion cells *in vivo*. J Mol Histol 2013; 44: 11–18. 23264107 10.1007/s10735-012-9446-7

[bib145] Dai M , Liu Y , Nie X , Zhang J , Wang Y , Ben J et al. Expression of RBMX in the light-induced damage of rat retina in vivo. Cell Mol Neurobiol 2015; 35: 463–471. 25407628 10.1007/s10571-014-0140-z PMC11486204

[bib146] Xu Y , Yang L , Yu SS , Shu QM , Yang C , Wang JW et al. Spatiotemporal changes in NFATc4 expression of retinal ganglion cells after light-induced damage. J Mol Neurosci 2014; 53: 69–77. 24362677 10.1007/s12031-013-0198-y

[bib147] Osborne NN , Casson RJ , Wood JP , Chidlow G , Graham M , Melena J . Retinal ischemia: mechanisms of damage and potential therapeutic strategies. Prog Retin Eye Res 2004; 23: 91–147. 14766318 10.1016/j.preteyeres.2003.12.001

[bib148] Buchi ER , Suivaizdis I , Fu J . Pressure-induced retinal ischemia in rats: an experimental model for quantitative study. Ophthalmologica 1991; 203: 138–147. 1775302 10.1159/000310240

[bib149] Hughes WF . Quantitation of ischemic damage in the rat retina. Exp Eye Res 1991; 53: 573–582. 1743256 10.1016/0014-4835(91)90215-z

[bib150] Yamamoto H , Schmidt-Kastner R , Hamasaki DI , Yamamoto H , Parel JM . Complex neurodegeneration in retina following moderate ischemia induced by bilateral common carotid artery occlusion in Wistar rats. Exp Eye Res 2006; 82: 767–779. 16359664 10.1016/j.exer.2005.09.019

[bib151] Lafuente MP , Villegas-Perez MP , Selles-Navarro I , Mayor-Torroglosa S , Miralles de Imperial J , Vidal-Sanz M . Retinal ganglion cell death after acute retinal ischemia is an ongoing process whose severity and duration depends on the duration of the insult. Neuroscience 2002; 109: 157–168. 11784707 10.1016/s0306-4522(01)00458-4

[bib152] Singh M , Savitz SI , Hoque R , Gupta G , Roth S , Rosenbaum PS et al. Cell-specific caspase expression by different neuronal phenotypes in transient retinal ischemia. J Neurochem 2001; 77: 466–475. 11299309 10.1046/j.1471-4159.2001.00258.x

[bib153] Kurokawa T , Katai N , Shibuki H , Kuroiwa S , Kurimoto Y , Nakayama C et al. BDNF diminishes caspase-2 but not c-Jun immunoreactivity of neurons in retinal ganglion cell layer after transient ischemia. Invest Ophthalmol Vis Sci 1999; 40: 3006–3011. 10549664

[bib154] Kurokawa T , Katai N , Kuroiwa S , Shibuki H , Kurimoto Y , Yoshimura N . BDNF suppresses expression of caspase-2 but not of c-Jun in rat retinal ganglion cells after ischemia-reperfusion injury. Invest Ophthalmol Vis Sci 1999; 40: S481. 10549664

[bib155] Lam TT , Abler AS , Tso MO . Apoptosis and caspases after ischemia-reperfusion injury in rat retina. Invest Ophthalmol Vis Sci 1999; 40: 967–975. 10102294

[bib156] Ishikawa S , Hirata A , Nakabayashi J , Iwakiri R , Okinami S . Neuroprotective effect of small interfering RNA targeted to caspase-3 on rat retinal ganglion cell loss induced by ischemia and reperfusion injury. Curr Eye Res 2012; 37: 907–913. 22642649 10.3109/02713683.2012.688161

[bib157] Zhang Z , Tong N , Gong Y , Qiu Q , Yin L , Lv X et al. Valproate protects the retina from endoplasmic reticulum stress-induced apoptosis after ischemia-reperfusion injury. Neurosci Lett 2011; 504: 88–92. 21939735 10.1016/j.neulet.2011.09.003

[bib158] Dvoriantchikova G , Ivanov D , Barakat D , Grinberg A , Wen R , Slepak VZ et al. Genetic ablation of Pannexin1 protects retinal neurons from ischemic injury. PLoS ONE 2012; 7: e31991. 22384122 10.1371/journal.pone.0031991 PMC3285635

[bib159] Niyadurupola N , Sidaway P , Ma N , Rhodes JD , Broadway DC , Sanderson J . P2X7 receptor activation mediates retinal ganglion cell death in a human retina model of ischemic neurodegeneration. Invest Ophthalmol Vis Sci 2013; 54: 2163–2170. 23449724 10.1167/iovs.12-10968

[bib160] Zhang Y , Cho CH , Atchaneeyasakul L , McFarland T , Appukuttan B , Stout JT . Activation of the mitochondrial apoptotic pathway in a rat model of central retinal artery occlusion. Invest Ophthalmol Vis Sci 2005; 46: 2133–2139. 15914634 10.1167/iovs.04-1235

[bib161] Shabanzadeh AP , D'Onofrio PM , Monnier PP , Koeberle PD . Targeting caspase-6 and caspase-8 to promote neuronal survival following ischemic stroke. Cell Death Dis 2015; 6: e1967. 26539914 10.1038/cddis.2015.272 PMC4670918

[bib162] Quigley HA , Broman AT . The number of people with glaucoma worldwide in 2010 and 2020. Brit J Ophthalmol 2006; 90: 262–267. 16488940 10.1136/bjo.2005.081224 PMC1856963

[bib163] Wolfs RC , Klaver CC , Ramrattan RS , van Duijn CM , Hofman A , de Jong PT . Genetic risk of primary open-angle glaucoma. Population-based familial aggregation study. Arch Ophthalmol 1998; 116: 1640–1645. 9869795 10.1001/archopht.116.12.1640

[bib164] Mukesh BN , McCarty CA , Rait JL , Taylor HR . Five-year incidence of open-angle glaucoma: the visual impairment project. Ophthalmology 2002; 109: 1047–1051. 12045042 10.1016/s0161-6420(02)01040-0

[bib165] Jha P , Banda H , Tytarenko R , Bora PS , Bora NS . Complement mediated apoptosis leads to the loss of retinal ganglion cells in animal model of glaucoma. Mol Immunol 2011; 48: 2151–2158. 21821293 10.1016/j.molimm.2011.07.012 PMC3653641

[bib166] Husain S , Abdul Y , Crosson CE . Preservation of retina ganglion cell function by morphine in a chronic ocular-hypertensive rat model. Invest Ophthalmol Vis Sci 2012; 53: 4289–4298. 22661469 10.1167/iovs.12-9467 PMC3392012

[bib167] Liu HX , Sun H , Liu CY . Interference of the apoptotic signaling pathway in RGC stress response by SP600125 in moderate ocular hypertensive rats. Chin J Physiol 2011; 54: 124–132. 21789894

[bib168] Hill LJ , Mead B , Blanch RJ , Ahmed Z , De Cogan F , Morgan-Warren PJ et al. Decorin reduces intraocular pressure and retinal ganglion cell loss in rodents through fibrolysis of the scarred trabecular meshwork. Invest Ophthalmol Vis Sci 2015; 56: 3743–3757. 26066743 10.1167/iovs.14-15622

[bib169] Taylor AW . Primary open-angle glaucoma: a transforming growth factor-beta pathway-mediated disease. Am J Pathol 2012; 180: 2201–2204. 22525463 10.1016/j.ajpath.2012.03.011 PMC3378912

[bib170] Levkovitch-Verbin H , Waserzoog Y , Vander S , Makarovsky D , Piven I . Minocycline upregulates pro-survival genes and downregulates pro-apoptotic genes in experimental glaucoma. Graef Arch Clin Exp 2014; 252: 761–772. 10.1007/s00417-014-2588-4 24566901

[bib171] McKinnon SJ , Lehman DM , Kerrigan-Baumrind LA , Merges CA , Pease ME , Kerrigan DF et al. Caspase activation and amyloid precursor protein cleavage in rat ocular hypertension. Invest Ophthalmol Vis Sci 2002; 43: 1077–1087. 11923249

[bib172] Huang W , Dobberfuhl A , Filippopoulos T , Ingelsson M , Fileta JB , Poulin NR et al. Transcriptional up-regulation and activation of initiating caspases in experimental glaucoma. Am J Pathol 2005; 167: 673–681. 16127148 10.1016/S0002-9440(10)62042-1 PMC1698740

[bib173] Fu QL , Li X , Shi J , Xu G , Wen W , Lee DH et al. Synaptic degeneration of retinal ganglion cells in a rat ocular hypertension glaucoma model. Cell Mol Neurobiol 2009; 29: 575–581. 19172389 10.1007/s10571-009-9349-7 PMC11505815

[bib174] Kim HS , Park CK . Retinal ganglion cell death is delayed by activation of retinal intrinsic cell survival program. Brain Res 2005; 1057: 17–28. 16139821 10.1016/j.brainres.2005.07.005

[bib175] Ji JZ , Chang P , Pennesi ME , Yang Z , Zhang J , Li DQ et al. Effects of elevated intraocular pressure on mouse retinal ganglion cells. Vis Res 2005; 45: 169–179. 15581918 10.1016/j.visres.2004.08.008

[bib176] Hanninen VA , Pantcheva MB , Freeman EE , Poulin NR , Grosskreutz CL . Activation of caspase 9 in a rat model of experimental glaucoma. Curr Eye Res 2002; 25: 389–395. 12789547 10.1076/ceyr.25.6.389.14233

[bib177] Chi W , Li F , Chen H , Wang Y , Zhu Y , Yang X et al. Caspase-8 promotes NLRP1/NLRP3 inflammasome activation and IL-1beta production in acute glaucoma. Proc Natl Acad Sci USA 2014; 111: 11181–11186. 25024200 10.1073/pnas.1402819111 PMC4121847

[bib178] Chi W , Chen H , Li F , Zhu Y , Yin W , Zhuo Y . HMGB1 promotes the activation of NLRP3 and caspase-8 inflammasomes via NF-kappaB pathway in acute glaucoma. J Neuroinflammation 2015; 12: 137. 26224068 10.1186/s12974-015-0360-2 PMC4518626

[bib179] Joachim SC , Mondon C , Gramlich OW , Grus FH , Dick HB . Apoptotic retinal ganglion cell death in an autoimmune glaucoma model is accompanied by antibody depositions. J Mol Neurosci 2014; 52: 216–224. 24091788 10.1007/s12031-013-0125-2

[bib180] Grus FH , Joachim SC , Hoffmann EM , Pfeiffer N . Complex autoantibody repertoires in patients with glaucoma. Mol Vis 2004; 10: 132–137. 14990890

[bib181] Joachim SC , Pfeiffer N , Grus FH . Autoantibodies in patients with glaucoma: a comparison of IgG serum antibodies against retinal, optic nerve, and optic nerve head antigens. Graefes Arch Clin Exp Ophthalmol 2005; 243: 817–823. 15834611 10.1007/s00417-004-1094-5

[bib182] Reichelt J , Joachim SC , Pfeiffer N , Grus FH . Analysis of autoantibodies against human retinal antigens in sera of patients with glaucoma and ocular hypertension. Curr Eye Res 2008; 33: 253–261. 18350436 10.1080/02713680701871157

[bib183] Joachim SC , Gramlich OW , Laspas P , Schmid H , Beck S , von Pein HD et al. Retinal ganglion cell loss is accompanied by antibody depositions and increased levels of microglia after immunization with retinal antigens. PLoS ONE 2012; 7: e40616. 22848388 10.1371/journal.pone.0040616 PMC3406064

[bib184] Ozdek S , Lonneville YH , Onol M , Yetkin I , Hasanreisoglu BB . Assessment of nerve fiber layer in diabetic patients with scanning laser polarimetry. Eye (Lond) 2002; 16: 761–765. 12439673 10.1038/sj.eye.6700207

[bib185] Takahashi H , Goto T , Shoji T , Tanito M , Park M , Chihara E . Diabetes-associated retinal nerve fiber damage evaluated with scanning laser polarimetry. Am J Ophthalmol 2006; 142: 88–94. 16815255 10.1016/j.ajo.2006.02.016

[bib186] Chihara E , Zhang S . [Analysis of diabetic optic neuropathy with a topographic laser scanning system]. Nippon Ganka Gakkai Zasshi 1998; 102: 431–435. 9720364

[bib187] Barber AJ , Lieth E , Khin SA , Antonetti DA , Buchanan AG , Gardner TW et al. Neural apoptosis in the retina during experimental and human diabetes - early onset and effect of insulin. J Clin Invest 1998; 102: 783–791. 9710447 10.1172/JCI2425 PMC508941

[bib188] Abu El-Asrar AM , Dralands L , Missotten L , Al-Jadaan I , Geboes K . Expression of apoptosis markers in the retinas of human subjects with diabetes. Invest Ophthalmol Vis Sci 2004; 45: 2760–2766. 15277502 10.1167/iovs.03-1392

[bib189] Oshitari T , Yamamoto S , Hata N , Roy S . Mitochondria- and caspase-dependent cell death pathway involved in neuronal degeneration in diabetic retinopathy. Brit J Ophthalmol 2008; 92: 552–556. 18369072 10.1136/bjo.2007.132308

[bib190] Barber AJ , Antonetti DA , Kern TS , Reiter CEN , Soans RS , Krady JK et al. The Ins2(Akita) mouse as a model of early retinal complications in diabetes. Invest Ophthalmol Vis Sci 2005; 46: 2210–2218. 15914643 10.1167/iovs.04-1340

[bib191] Gastinger MJ , Kunselman AR , Conboy EE , Bronson SK , Barber AJ . Dendrite remodeling and other abnormalities in the retinal ganglion cells of Ins2(Akita) diabetic mice. Invest Ophthalmol Vis Sci 2008; 49: 2635–2642. 18515593 10.1167/iovs.07-0683

[bib192] Martin PM , Roon P , Van Ells TK , Ganapathy V , Smith SB . Death of retinal neurons in streptozotocin-induced diabetic mice. Invest Ophthalmol Vis Sci 2004; 45: 3330–3336. 15326158 10.1167/iovs.04-0247

[bib193] Yang JH , Kwak HW , Kim TG , Han J , Moon SW , Yu SY . Retinal neurodegeneration in type II diabetic Otsuka Long-Evans Tokushima fatty rats. Invest Ophthalmol Vis Sci 2013; 54: 3844–3851. 23640038 10.1167/iovs.12-11309

[bib194] Mohr S , Xi X , Tang J , Kern TS . Caspase activation in retinas of diabetic and galactosemic mice and diabetic patients. Diabetes 2002; 51: 1172–1179. 11916941 10.2337/diabetes.51.4.1172

[bib195] Hernandez C , Garcia-Ramirez M , Corraliza L , Fernandez-Carneado J , Farrera-Sinfreu J , Ponsati B et al. Topical administration of somatostatin prevents retinal neurodegeneration in experimental diabetes. Diabetes 2013; 62: 2569–2578. 23474487 10.2337/db12-0926 PMC3712066

[bib196] Li YH , Zhuo YH , Lu L , Chen LY , Huang XH , Zhang JL et al. Caspase-dependent retinal ganglion cell apoptosis in the rat model of acute diabetes. Chin Med J (Engl) 2008; 121: 2566–2571. 19187597

[bib197] Oshitari T , Yoshida-Hata N , Yamamoto S . Effect of neurotrophic factors on neuronal apoptosis and neurite regeneration in cultured rat retinas exposed to high glucose. Brain Res 2010; 1346: 43–51. 20573599 10.1016/j.brainres.2010.05.073

[bib198] Hu Y , Park KK , Yang L , Wei X , Yang Q , Cho KS et al. Differential effects of unfolded protein response pathways on axon injury-induced death of retinal ganglion cells. Neuron 2012; 73: 445–452. 22325198 10.1016/j.neuron.2011.11.026 PMC3278720

[bib199] Koeberle PD , Wang Y , Schlichter LC . Kv1.1 and Kv1.3 channels contribute to the degeneration of retinal ganglion cells after optic nerve transection *in vivo*. Cell Death Differ 2010; 17: 134–144. 19696788 10.1038/cdd.2009.113

[bib200] Kim SY , Shim MS , Kim KY , Weinreb RN , Wheeler LA , Ju WK . Inhibition of cyclophilin D by cyclosporin A promotes retinal ganglion cell survival by preventing mitochondrial alteration in ischemic injury. Cell Death Dis 2014; 5: e1105. 24603333 10.1038/cddis.2014.80 PMC3973219

[bib201] Levkovitch-Verbin H , Waserzoog Y , Vander S , Makarovsky D , Ilia P . Minocycline mechanism of neuroprotection involves the Bcl-2 gene family in optic nerve transection. Int J Neurosci 2014; 124: 755–761. 24410139 10.3109/00207454.2013.878340

[bib202] Zhou W , Zhu X , Zhu L , Cui YY , Wang H , Qi H et al. Neuroprotection of muscarinic receptor agonist pilocarpine against glutamate-induced apoptosis in retinal neurons. Cell Mol Neurobiol 2008; 28: 263–275. 18172757 10.1007/s10571-007-9251-0 PMC11516528

[bib203] Inomata Y , Nakamura H , Tanito M , Teratani A , Kawaji T , Kondo N et al. Thioredoxin inhibits NMDA-induced neurotoxicity in the rat retina. J Neurochem 2006; 98: 372–385. 16805832 10.1111/j.1471-4159.2006.03871.x

[bib204] Sun C , Li XX , He XJ , Zhang Q , Tao Y . Neuroprotective effect of minocycline in a rat model of branch retinal vein occlusion. Exp Eye Res 2013; 113: 105–116. 23748101 10.1016/j.exer.2013.05.018

[bib205] Yuan D , Xu Y , Hang H , Liu X , Chen X , Xie P et al. Edaravone protect against retinal damage in streptozotocin-induced diabetic mice. PLoS ONE 2014; 9: e99219. 24897298 10.1371/journal.pone.0099219 PMC4045952

[bib206] Wang Y , Zhang H , Liu Y , Li P , Cao Z , Cao Y . Erythropoietin (EPO) protects against high glucose-induced apoptosis in retinal ganglional cells. Cell Biochem Biophys 2015; 71: 749–755. 25287674 10.1007/s12013-014-0259-z

[bib207] Cao Y , Li X , Shi P , Wang LX , Sui ZG . Effects of L-carnitine on high glucose-induced oxidative stress in retinal ganglion cells. Pharmacology 2014; 94: 123–130. 25247444 10.1159/000363062

